# Study protocol for a two-site clinical trial to validate a smartphone-based artificial intelligence classifier identifying cervical precancer and cancer in HPV-positive women in Cameroon

**DOI:** 10.1371/journal.pone.0260776

**Published:** 2021-12-16

**Authors:** Inès Baleydier, Pierre Vassilakos, Roser Viñals, Ania Wisniak, Bruno Kenfack, Jovanny Tsuala Fouogue, George Enownchong Enow Orock, Sophie Lemoupa Makajio, Evelyn Foguem Tincho, Manuela Undurraga, Magali Cattin, Solomzi Makohliso, Klaus Schönenberger, Alain Gervaix, Jean-Philippe Thiran, Patrick Petignat

**Affiliations:** 1 University of Geneva, Faculty of Medicine, Geneva, Switzerland; 2 Department of Pediatrics, Gynecology and Obstetrics, University Hospitals of Geneva, Geneva, Switzerland; 3 Signal Processing Laboratory (LTS5), École Polytechnique Fédérale de Lausanne (EPFL), Lausanne, Switzerland; 4 Department of Obstetrics Gynecology and Maternal Health, Dschang District Hospital, University of Dschang, Dschang, Cameroon; 5 Regional Hospital of Bafoussam, Bafoussam, Cameroon; 6 EssentialTech Centre, École Polytechnique Fédérale de Lausanne (EPFL), Lausanne, Switzerland; Karolinska Institutet, SWEDEN

## Abstract

**Introduction:**

Cervical cancer remains a major public health challenge in low- and middle-income countries (LMICs) due to financial and logistical issues. WHO recommendation for cervical cancer screening in LMICs includes HPV testing as primary screening followed by visual inspection with acetic acid (VIA) and treatment. However, VIA is a subjective procedure dependent on the healthcare provider’s experience. Its accuracy can be improved by computer-aided detection techniques. Our aim is to assess the performance of a smartphone-based Automated VIA Classifier (AVC) relying on Artificial Intelligence to discriminate precancerous and cancerous lesions from normal cervical tissue.

**Methods:**

The AVC study will be nested in an ongoing cervical cancer screening program called “3T-study” (for Test, Triage and Treat), including HPV self-sampling followed by VIA triage and treatment if needed. After application of acetic acid on the cervix, precancerous and cancerous cells whiten more rapidly than non-cancerous ones and their whiteness persists stronger overtime. The AVC relies on this key feature to determine whether the cervix is suspect for precancer or cancer. In order to train and validate the AVC, 6000 women aged 30 to 49 years meeting the inclusion criteria will be recruited on a voluntary basis, with an estimated 100 CIN2+, calculated using a confidence level of 95% and an estimated sensitivity of 90% +/-7% precision on either side. Diagnostic test performance of AVC test and two current standard tests (VIA and cytology) used routinely for triage will be evaluated and compared. Histopathological examination will serve as reference standard. Participants’ and providers’ acceptability of the technology will also be assessed. The study protocol was registered under ClinicalTrials.gov (number NCT04859530).

**Expected results:**

The study will determine whether AVC test can be an effective method for cervical cancer screening in LMICs.

## Introduction

Cervical cancer remains one of the leading causes of mortality in low- and some medium-income countries (LMICs), where approximately 90% of its global burden is found [1]. This cancer mainly appears several years after a sexually-transmitted infection with HPV. Due to limited resources and infrastructures, the implementation of organized screening and vaccination programs at national levels remains a challenge in LMICs [2]. Yet, the necessity of cost-effective measures is clear as the global burden of cervical cancer is projected to significantly increase by 2030: new cases should rise from 570’000 in 2018 to nearly 700,000. In the same trend, the annual number of deaths could reach 400’000 by 2030, which represents an increase of 25% compared to 2018 [3]. In view of these projections, the World Health Organization (WHO) declared cervical cancer a major public health problem and elaborated a global strategy towards its eradication [4].

To achieve this goal, three targets need to be met by 2030 according to the “WHO 90-70-90” strategy: 90% of girls fully vaccinated by age 15, 70% of women screened with a high-performance test twice during each woman life’s time (by 35 and again by 45 years of age), and 90% of women identified with cervical precancer and cancer receiving appropriate treatment and care [4]. WHO recommends primary HPV testing as preferred method for screening women over 30 years of age living in LMICs [5]. The high sensitivity and negative predictive value of HPV testing allows large screening intervals of five years or more [6]. Furthermore, some diagnostic devices provide rapid point-of-care HPV testing analyzing self-obtained vaginal samples and allowing immediate treatment [7,8]. These advantages improve the cost-effectiveness and coverage of screening programs.

However, HPV testing used as a stand-alone test has a limited specificity and positive predictive value, as most infections tend to resolve spontaneously overtime. Consequently, a significant number of HPV-positive women receive unnecessary workup and potentially treatment. For this reason, the WHO recommends triage by visual inspection with acetic acid (VIA) of HPV-positive women to precisely identify women requiring treatment [5].

Initially, VIA was widely promoted and routinely used as a low-cost primary screening test in LMIC context. This procedure is straightforward and can be performed by a variety of trained health workers including midwives and nurses: a 3–5% acetic acid solution is applied on the cervix and the appearance of acetowhite areas define areas of the cervix compatible with precancerous or cancerous lesions. Nevertheless, VIA remains a subjective method highly variable and dependent on the health care providers [9–12]. Its reported sensitivity for detecting CIN2+ lesions varies between 25 and 80% between studies [13,14]. Therefore, an objective approach based on quantitative diagnostic algorithms is desirable to improve performance of VIA. Tools assisting front-line providers should be affordable, non-invasive and reliable.

With this objective and in a collaboration between the Gynecology and Obstetrics Department of the Geneva University Hospital (HUG) and the Swiss Federal Institute of Technology in Lausanne (EPFL), our group started the development of an automated smartphone-based image classification device called Automatic VIA Classifier (AVC). Two-minute videos of the cervix are recorded during VIA and classified using an artificial neural network (ANN) and image processing techniques to differentiate precancer and cancer from non-neoplastic cervical tissue.

The aim of this paper is to describe a protocol of a two-site clinical trial destined to finalize the classifier and determine its diagnostic performance for the triage of HPV-positive women in a low-resource context.

## Materials and methods

### AVC test–process of automatic detection and classification

**Fig 1:**
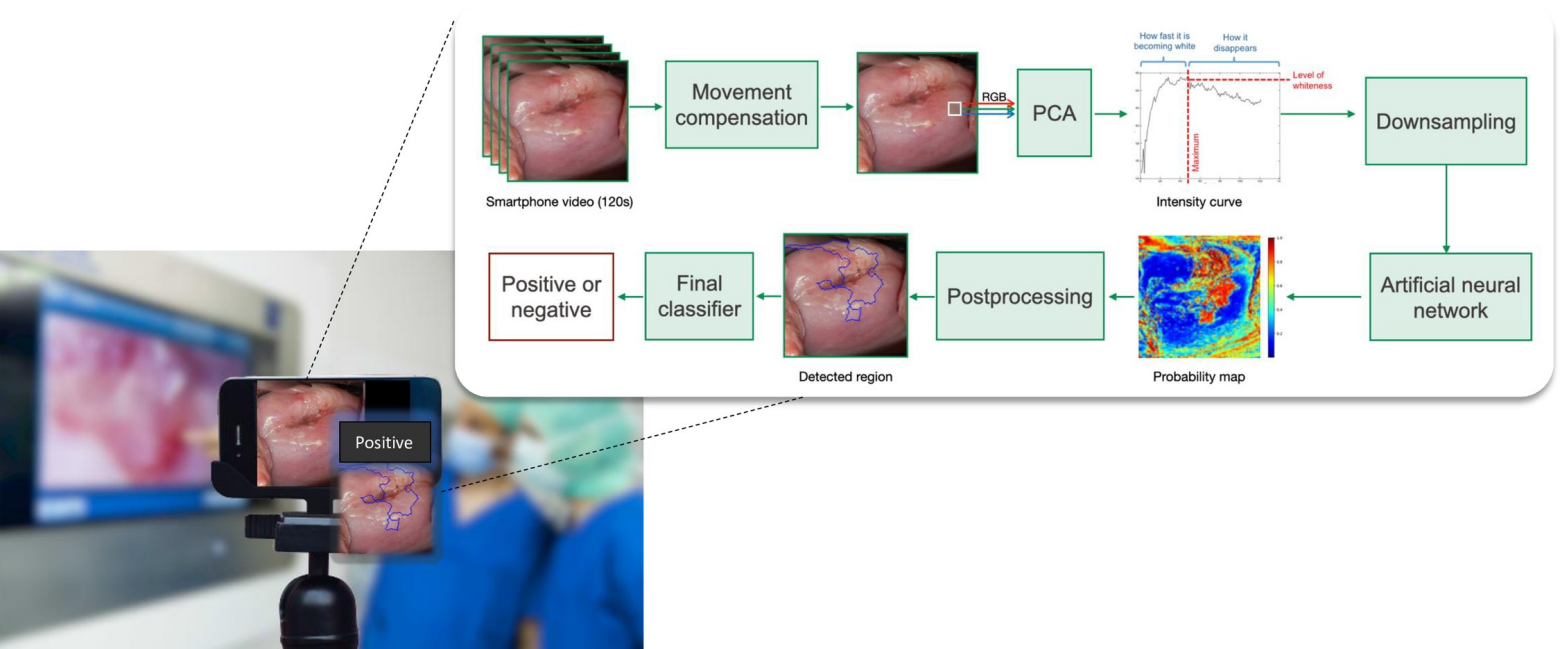
Process description of the AVC test. Adapted with permission from [15].

The key feature used by the classifier is the dynamic whitening of cervical tissue after application of acetic acid. While healthy regions remain light pink, dysplastic cells whiten rapidly as they have a high concentration of nuclear proteins and cytokeratins; the whiteness then decreases smoothly. Below, we detail the process of automatic detection and classification (Fig 1).

1. 120s videos at 1 frame per second (fps) are recorded by a smartphone during VIA. Thus, 120 frames are obtained.
2. Motion compensation is applied to stabilize the video.
3. For each pixel, Principal Component Analysis (PCA) is used to convert each image of the video from a three-dimensional RGB (red, green, blue) color space into a one-dimensional space with a single channel.
4. The resulting intensity curve of each pixel overtime is downsampled to 12 time-points. The first sample corresponding to the 10 first seconds of the video is discarded as it is the most affected by residual movement.
5. Each pixel, represented by its 11 samples, is independently given as input to an ANN, which contains one hidden layer. Thus, the ANN performs the classification pixel-wise.
6. As an output, the ANN provides the probability of the input pixel of being positive (precancer or cancer). The probabilities of all pixels of one image are combined in a probability map.
7. The probability map is postprocessed by applying region growing segmentation followed by a closing operation. Then, the contours of the lesions are obtained, delimitating all predicted lesions. The size of the lesions is compared to a predefined threshold (450 pixels) to decide whether the patient is negative or positive [15].
8. At the end, the test result (positive/negative) is displayed on the smartphone screen and when positive, predicted lesions are delimitated to assist biopsy if needed.

#### Preliminary results

Preliminary results indicate that the accuracy of the algorithm was 0.89. The precision, sensitivity, and specificity achieved were 0.93, 0.90, and 0.87, respectively [15]. These results were obtained based on videos from 44 patients. We expect to further improve the performance of the algorithm by increasing the amount of data in the near future.

### AVC study—objectives, endpoints and hypothesis

#### Primary objective

To determine the diagnostic performance of the AVC test used as a triage test.

#### Secondary objectives

To compare diagnostic performances of the AVC test with the current triage tests (VIA and cytology).

To assess the feasibility of integrating the AVC test in a cervical cancer screening program in a low-resource context.

To assess the acceptability of the AVC test as an innovative screening method by women and health care providers.

To reveal the main factors leading to discrepancies in the feasibility and acceptability of the AVC test between two clinical sites.

#### Primary endpoint

Estimate accuracy of the AVC test by including metrics such as sensitivity, specificity, positive predictive value and negative predictive value using histologic assessment as reference standard.

#### Secondary endpoints

Compare test accuracies between AVC test, VIA and cytology.

Estimate feasibility and acceptability of the AVC test by women and healthcare providers using qualitative and quantitative methods.

#### Ancillary studies

Cost effectiveness evaluation.

Estimation of overtreatment & clinical complications associated with screening process and treatment.

#### Hypotheses

(i) The AVC test identifies the majority of precancers and cancers among HPV-positive women. (ii) Its performance exceeds those obtained by cytology and VIA. (iii) The method is feasible and acceptable for women and health providers.

### Study design

The AVC study is a prospective two-site clinical trial that will be nested in a large prospective trial named *3T study* started in 2018 in Cameroon, after the implementation of the 3T screening program (Test-Triage-Treat in one day visit). This program results from the close collaboration between the Universities of Yaoundé, Dschang and Geneva who have been working together since 1997 to implement WHO guidance on cervical cancer control. Women participating in the *3T-Study* will be invited to participate in the *AVC study*.

#### Inclusion criteria

Women aged 30–49 years.

Free and informed consent to take part in the study on a voluntary basis.

#### Exclusion criteria

Pregnancy at the screening consultation.

Any condition altering the cervix visualization at the screening consultation (e.g. heavy vaginal bleeding).

History of anogenital cancer or known anogenital cancer at the screening consultation.

Previous hysterectomy.

Not sufficiently healthy to participate in the study.

#### Recruitment

Recruitment will take place in two distinct health districts of the West Region of Cameroon: the Dschang Health District (approximately 230’000 inhabitants) and Mifi Health District (approximately 370’000 inhabitants). There will be two screening centers: one in Dschang District Hospital for Dschang Health District and another in Bafoussam Regional Hospital for Mifi Health District. Both Health Districts have an estimated number of 70’000 women aged 30 to 49 years and potentially eligible for the *AVC study*. Recruitment announcements will specify the eligibility criteria, place and time of the screening. The free-nature of the screening program will be emphasized. Information will be spread through local radio announcements, hospital and public spaces banners, in churches and women’s associations. Community health workers will also be specially trained to recruit participants in their dedicated health sector.

#### Participant information and informed consent

The investigators will explain to participants the nature of the study, its purpose, the procedures involved, the expected duration, the potential risks and benefits and any discomfort it may entail. Each participant will be informed that the participation in the study is voluntary and that she may withdraw from the study at any time and that withdrawal of consent will not affect her subsequent medical assistance and treatment. The participant will be informed that her medical records may be examined by authorized individuals other than their treating physician.

All participants of the study will be provided a participant information sheet and a consent form describing the study and providing sufficient information for the participant to make an informed decision about their participation in the study. A patient information sheet and a consent form reviewed and approved by the Competent Ethics Committee (CEC) will be used. The formal consent of a participant, using the approved consent form, must be obtained before the participant is submitted to any study procedure.

The participant should read and consider the statement before signing and dating the informed consent form and should be given a copy of the signed document. In case of analphabetism, the consent form will be orally explained and signed with a X mark letter. The consent form must also be signed and dated by the investigator (or his designee) and it will be retained as part of the study records.

#### Study procedure

**Fig 2:**
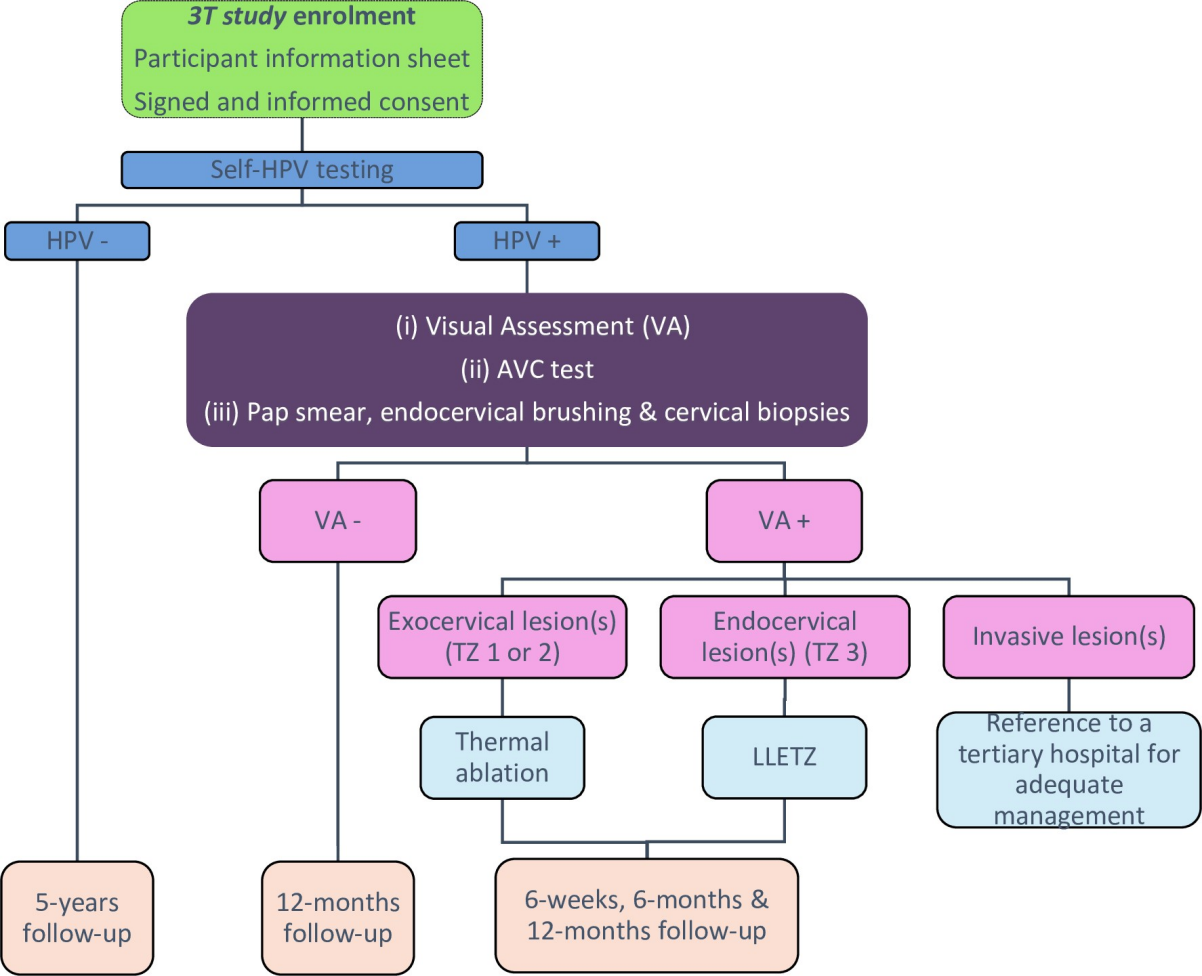
Flowchart of the clinical trial. Abbreviations: TZ = Transformation zone, LLETZ = Large loop excision of the transformation zone.

The *AVC study* will be integrated in the *3T study* as follows (Fig 2):

1. Prior to the enrollment of participants, a training course about the screening procedure with integration of the AVC test will be organized for local midwives and on-site personnel. Updated manuals (guidelines) will be distributed.
2. Trained midwives will share relevant information about cervical cancer and sexually transmitted diseases to women meeting the inclusion criteria of the *3T study*. They will present the *AVC study* (nature, purpose, procedures) in oral and written forms. Voluntary consents will be signed.
3. Sociodemographic data and medical parameters will be collected on a previously validated questionnaire.
4. Women will be invited to carry out an HPV self-test using a vaginal swab. The swab will be rinsed in a vial with 20 ml of saline solution (sodium chloride 0.9%), vortexed for 30 seconds, and transferred into a single-use disposable cartridge that holds polymerase chain reaction (PCR) reagents of the GeneXpert® analyzer (Cepheid®). The Xpert system provides quality results in 60 minutes. Specifically, it uses five color channels containing primers and probes for the detection of the following specific genotypes or pooled results: i) HPV 16, ii) HPV 18, 45 in a pooled result, iii) HPV types 31, 33, 35 52, or 58, in a pooled result, iv) HPV types 51 or 59, in a pooled result, and v) HPV types 39, 56, 66 or 68 in a pooled result.
5. HPV-negative women will receive counseling and recommendations to repeat screening after 5 years. HPV-positive women will be invited to undergo a gynecological examination by the respondent midwife including visual inspection with acetic acid (VIA) and with Lugol’s iodine (VILI), the AVC test and histopathological sampling by the respondent midwife as described below.
6. VIA/VILI will be carried out by a nurse who will first apply a cotton swab soaked with acetic acid on the cervix. The result will be evaluated after waiting one minute and the same procedure will be repeated with iodine to confirm the findings and facilitate treatment.
7. Digital images of the cervix during the VIA/VILI procedure (*D-VIA/VILI)* will be performed with a smartphone application named “Exam” to obtain high-quality digital pictures [16]. D-VIA/VILI are used as adjuncts to naked-eye inspection of VIA/VILI for final diagnosis. They are also used by expert colposcopists for quality control, experience sharing and teaching [16–18]. For clarity, combined VIA/VILI and D-VIA/VILI are referred to as Visual Assessment (VA) hereafter. We introduced “relaxed WHO criteria” for the interpretation of VA: any aceto-white lesion ≥0.5 cm localized in the transformation zone, including faint whitening, is considered VA positive and referred for treatment. We implemented an ABCD mnemonic method to alert health providers for positivity of VA. It consists of the following simple criteria: A for “Acetowhiteness”, B for “Bleeding of a lesion in the transformation zone”, C for “Coloring confirmation with Lugol’s Iodine” and D for “Diameter of the acetowhite area” (≥0.5 cm) [19].
8. The AVC test will be performed during the VA procedure: 120 second videos focused on the cervix will be taken right after the application of acetic acid. The recording smartphone will be fixed on a tripod situated 15cm away from the vulval vestibule.
9. HPV-positive women will have a liquid-based Pap test using the ThinPrep technique. A spatula will be used to collect cells from the transformation zone (TZ) of the cervix, and the cell‐covered end of the spatula will be introduced into a vial containing a preservative solution; cytological results will be classified according to the Bethesda classification system by qualified cytotechnologists. Cervical tissue from endocervical brushing and from cervical biopsies will be obtained for histopathological examination (reference standard). Biopsies will be performed on all lesions highlighted during VA. If no lesion is seen, a random biopsy at 6 o’clock within the transformation zone will be obtained. Cytological and histological samples will be prepared and diagnosed in the Division of Clinical Pathology (Geneva University Hospitals).
10. Therapy and follow-up will be based on the results of VA.
  - VA positive women will receive immediate treatment: thermal ablation will be used for fully accessible exocervical lesions situated in the TZ type 1 or 2. Inaccessible endocervical lesions (TZ 3) will be treated by large loop excision of the transformation zone (LLETZ). Treated women will be recalled for three follow-up visits. The first one will be planned at 4 to 6 weeks to monitor the occurrence of any adverse events or complication related to the treatment. A survey about the acceptability of the entire procedure will also be filled. Second and third follow-up visits will take place at 6 and 12 months, both involving a self-HPV test. If positive, they will undergo a gynecological consultation including VA, cytological and histopathological samplings as appropriate.
  - VA negative women will be recalled at 12 months for HPV testing, unless the histopathological and cytological results come back positive, in which case they will be rapidly recalled.
  - If invasive cancer is suspected, women will be referred to a tertiary hospital for appropriate clinical management.
11. At the end of the first visit, a survey about the acceptability of the screening procedure, the gynecological examination and the treatment, will be filled by each participant, if relevant.

#### Training phase and validation phase

The AVC trial will be divided in two phases. First, a training phase using recorded videos from 176 HPV-positive women will constitute the “training set” and will be transmitted to the EPFL for further training of the classifier and improvement of performance. Histopathological analyses will constitute the reference standard. Second, the upgraded version of the AVC test will be integrated in a mobile application designed by EPFL and used during the validation phase which will include 830 HPV-positive women. Recorded videos will constitute the “validation set” and will still be transmitted to the EPFL for quality control.

### Statistical analysis

The study will compare diagnostic accuracy to detect precancerous lesions and cancer among HPV-positive women of the AVC test, conventional VIA and cytology, used as stand-alone tests and in combination. Sensitivity, specificity, positive and negative predictive values, positive and negative likelihood ratios will be calculated with 95% confidence intervals. Diagnostic odds ratios with 95% confidence intervals and an estimated sensitivity of 90% +/-7% precision on either side will be calculated for comparison of diagnostic accuracies between triage tests. A receiver operating characteristic curve (ROC) will be determined (sensitivity against 1-specificity) to evaluate diagnostic ability. Calculations will be performed using a statistical analysis software package (Stata Corp: Release 16, College Station, TX, USA).

#### Reference standard

Histopathological results from biopsies and endocervical brushing will be used as the reference standard to evaluate accuracy in this study. Two pathologists specialized in gynecopathology, blinded from cytology results, will provide adjudicated diagnosis according to the CIN classification system.

#### Sample size calculation

**Training set:** this set will include the screening of 1000 Cameroonian women: from our experience, we expect approximately 17% of all screened women to be HPV-positive, and a 10% prevalence of CIN2+ cases in HPV-positive women. Thus, we will obtain 170 HPV-positive cases and 17 CIN2+ cases.

**Validation set:** using the formula n = (Z1−α2)2xSx(1‐S)d2xprevalence=1.962x0.9x0.10.072x0.1, where n = required sample size, S = anticipated sensitivity, α = test size, 1–α2 the confidence level set at 95%, Z1−α2 = 1.96 the corresponding standard normal deviate, d = planned anticipated width of the eventually calculated confidence interval, we calculated a sample size of 706 HPV-positive women.

We estimate 12–15% problems with processing and reading of HPV tests and biopsies. Therefore, a sample size of 5000 participants (~ 830 HPV-positive and 83 CIN2+) will be necessary.

### Data management plan

**Fig 3:**
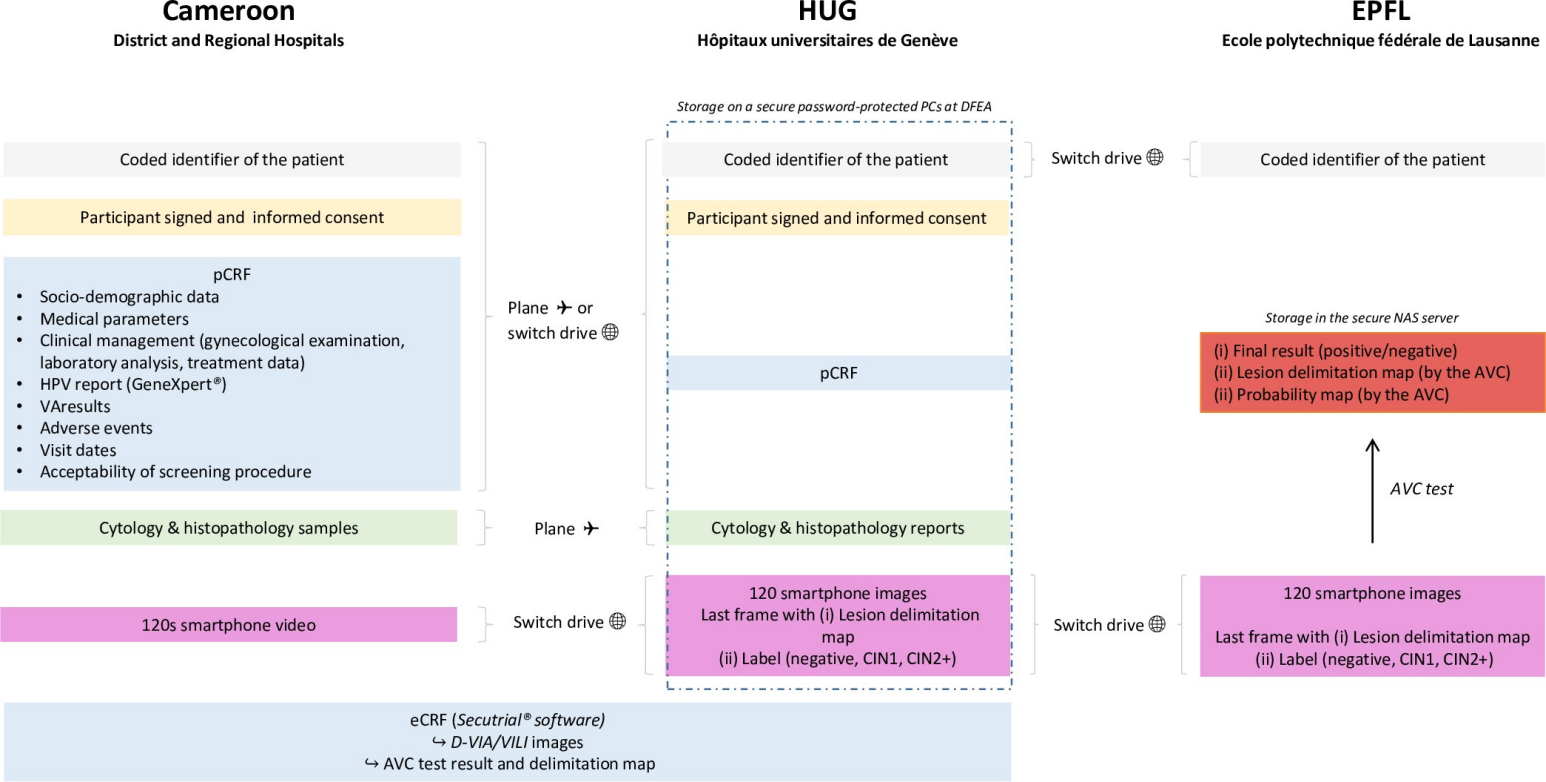
*3T study* data management plan. Cameroon: participants will be pseudonymized with an identification key consisting of a code number and the participant’s initials. A paper Case Report Form (pCRF) will be created for each research subject: it will contain its personal, medical and screening-related information. These data will also be electronically stored (eCRF) in a RDBMS (Relational Database Management System) using a dedicated CDMS software (Clinical Database Management System) called secuTrial®. This software will also be used to save VA images, AVC test results and delimitation maps. University Hospitals of Geneva: a secure password-protected PC at the Department of Pediatrics, Gynecology and Obstetrics (DFEA) will store all collected data (dashed rectangle) and eCRFs will be accessible on secuTrial®. Data management will be performed by the Unit of Clinical Investigation (UIC), a unit which is part of the Clinical Research Center (CRC) at HUG as well as by designated research assistants at the DFEA. Cytological and histopathological samples will be transported by aircraft and analyzed at the Division of Clinical Pathology of the HUG, in conformity with Swiss standards and international recommendations. Smartphone videos will be shared by SWITCH drive *(**https*:*//www*.*switch*.*ch/drive/**)* in the form of 120 frames. The last frame will be labelled by a colposcopy specialist of HUG who will also manually delimitate any visible lesion. EPFL: Final results (positive/negative), lesion delimitation maps and probability maps (PNG format) obtained during the AVC test will be stored in a secure NAS server in EPFL.

Data will be collected and transferred between District and Regional Hospitals in Cameroon, the HUG and the EPFL as shown below (Fig 3).

All study data will be archived in the CRC at HUG for a minimum of 10 years after study termination.

### Schedule and visits

#### Flow of the project: Schedule and milestones

**Fig 4:**
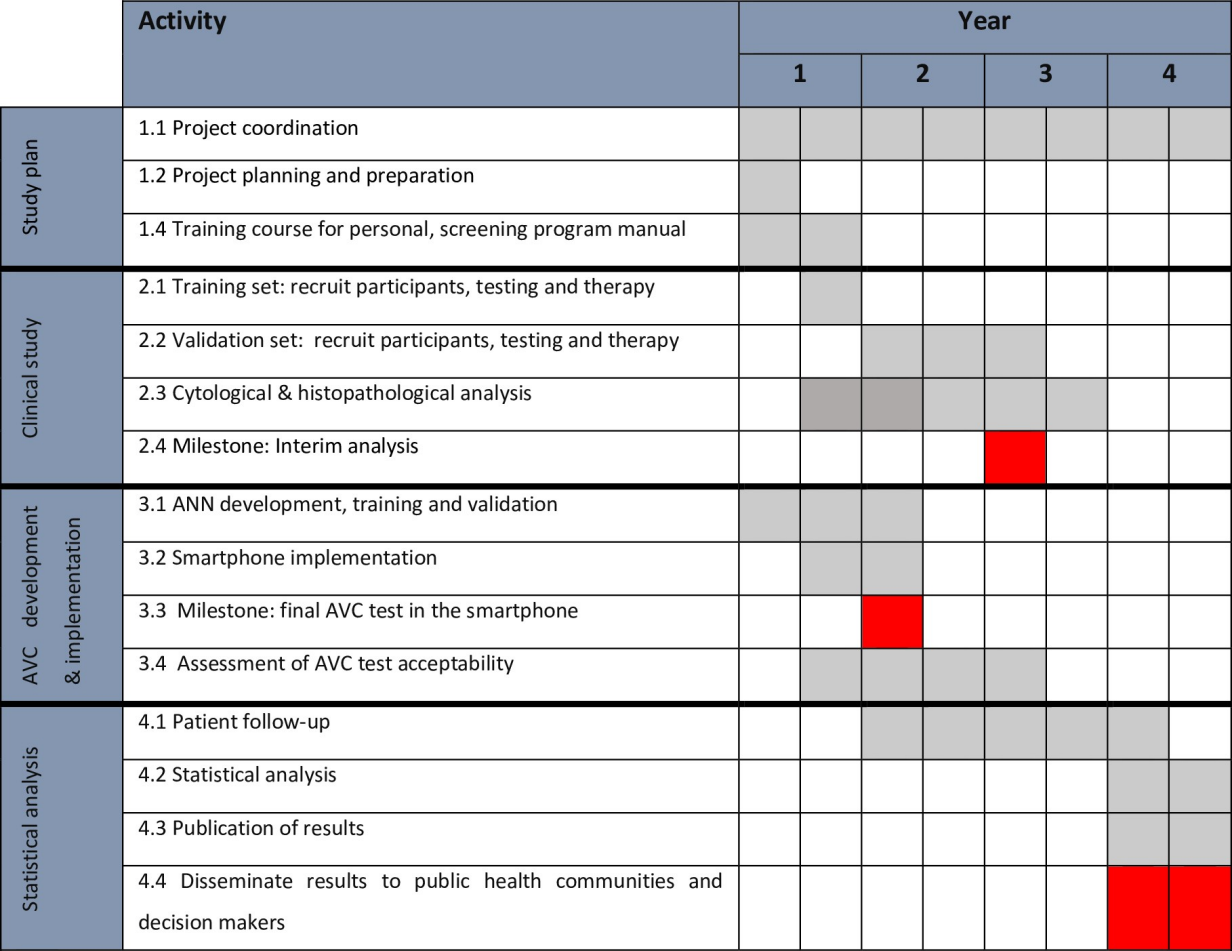
Project schedule and milestones.

The project will take place within a 4-year period. The work will be divided as follows (Fig 4).

#### Visits

Professional and scientific visits between Cameroon and Geneva, Switzerland, take place on a regular basis: resident physicians from HUG benefit from training rotations at the Dschang District Hospital, maintaining constant relations between Swiss and Cameroonian research teams. The principal investigator and co-investigators of the *3T study*, including responsible researchers for each study site, meet once a year in Cameroon for general supervision and monitoring of the study.

#### Usability and acceptance studies, project sustainability and deployment plan

We plan to (1) understand and define the optimal place to position the test in the health system and in the patient journey, in collaboration with the WHO and the relevant stakeholders in the pilot countries in sub-Saharan Africa, (2) design a local stakeholder engagement study in order to assess barriers and facilitators to implement and scale-up the test. Consultations will be undertaken with relevant stakeholders, including the relevant medical personnel and patients, (3) design a usability study to (a) assess the time required for the frontline health professionals to adequately integrate the AVC test within the screening procedure and (b) assess acceptance of the user interface of the application initially and the final screening test subsequently, (4) develop a social business model that will ensure that the deployment and scale-up of the test can be self-sustaining and have long-term viability.

In addition to the survey about the acceptability of the entire procedure given to the patients, the acceptability of the artificial intelligence-based technology will be studied via the organization of focus groups, involving stakeholders with various perspectives, i.e. healthcare providers, patients and community members at a wider scale such as patients’ partners.

Cost data have been collected since the start of the study regarding personnel remuneration, training and equipment costs. Further data are currently being collected regarding the time required for each step of the screening and treatment procedure.

### Ethical and safety considerations

#### Ethics committee

The protocol has obtained approval from the Cantonal Ethics Board of Geneva, Switzerland (Commission cantonale d’éthique de la recherche, N°2017–01110, 21/04/20) and the National Ethics Committee for Research on Human Health, Cameroon (Comité national d’éthique de la recherche pour la santé humaine, CNERSH, N°2018/07/1083/CE/CNERSH/SP, 24/07/18).

#### Risk categorization

The study’s risk category is A according to swiss ethical guidelines. This decision is based on the fact that the planned measures for sampling biological material or collecting personal data entail only minimal risks and burdens.

#### Participant privacy and safety

The investigator affirms and upholds the principle of the participants’ right to dignity, privacy and health and that the project team shall comply with applicable privacy laws. Especially, anonymity of the participants shall be guaranteed when presenting the data at scientific meetings or publishing them in scientific journals.

Individual subject medical information obtained as a result of this study is considered confidential and disclosure to third parties is prohibited. Subject confidentiality will be further ensured by utilizing subject identification code numbers to correspond to treatment data in the computer files.

For data verification purposes, authorized representatives of the Sponsor (-Investigator), a competent authority or an ethics committee may require direct access to parts of the medical records relevant to the study, including participants’ medical history.

#### External advisory board, dissemination, protocol and data availability

An independent external advisory board has been selected and composed by members of Cameroonian and Swiss medical community. Its responsibility includes providing advice on study validity and integrity, assure participant safety, suggest improvements or modifications to the procedures, and at the completion of the study, disseminate results to Cameroonian stakeholders. Results will also be disseminated to the scientific community through peer-reviewed journal articles, and international conference presentations. The protocol and anonymized data will be available upon request to the principal investigator of the study.

## Discussion

Artificial Intelligence is revolutionizing medical imaging and has demonstrated considerable potential in computer-assisted diagnosis. Previous studies using cervigrams to train and validate deep learning algorithms showed that algorithms using artificial intelligence can automatically distinguish normal and abnormal (CIN2+) cervical tissue after acetic acid application, thus improving diagnostic accuracy. However, most of these studies were performed in controlled settings and with selected datasets [20,21]. The proposed prospective *AVC study* will be conducted in a real-world setting and the algorithm will be tested in a real-life diagnosis process. Furthermore, all HPV-positive women will undergo biopsy and endocervical brushing to minimize disease misclassification. The results of this trial will represent an important piece of evidence showing how this technology can be integrated in a screening program. Through our planned cost-effectiveness analysis and the qualitative research, we will be able to make an assumption about the feasibility of our strategy.

Furthermore, we expect the described trial to confirm that the mean accuracy, precision, sensitivity and specificity of the AVC test outperforms human assessment of VIA, as shown in our preliminary results [15]. Evidence should be found that the AVC test allows a reliable automation of cervical precancer and cancer detection. Through its smartphone-based interface, the developed tool should meet the needs and means of LMICs: it is an affordable screening tool allowing an intuitive and user-friendly use by different health professionals, requiring only minimal training. Thus, the AVC test may be a key adjunct to any cervical cancer screening program in LMICs.

In a large-scale strategy, we believe our smartphone-based classifier will represent an innovative solution to a major public health problem in LMICs: it is in the top priorities of the WHO and of African decision-makers to reduce the evitable burden of cervical cancer in LMICs. In an even larger perspective, the AVC test could be extended to high-income countries, where both cytology and colposcopy are routinely used for triage of detected cervical abnormalities despite their poor reproducibility. Therefore, an AVC software could be used to classify cervical images taken with a colposcope with the goal to develop an objective approach based on a quantitative algorithm for a more reliable diagnosis.

## Supporting information

S1 Checklist SPIRIT 2013 checklist. (PDF)

S1 Fig SPIRIT schedule. (TIF)

S1 File Clinical study protocol NCT03757299. (PDF)
