# Supplementary figures and images for: Study protocol for a two-site clinical trial to validate a smartphone-based artificial intelligence classifier identifying cervical precancer and cancer in HPV-positive women in Cameroon
Source: PLoS One. 2021 Dec 16;16(12):e0260776. doi: 10.1371/journal.pone.0260776 (PMC8675688; doi:10.1371/journal.pone.0260776)

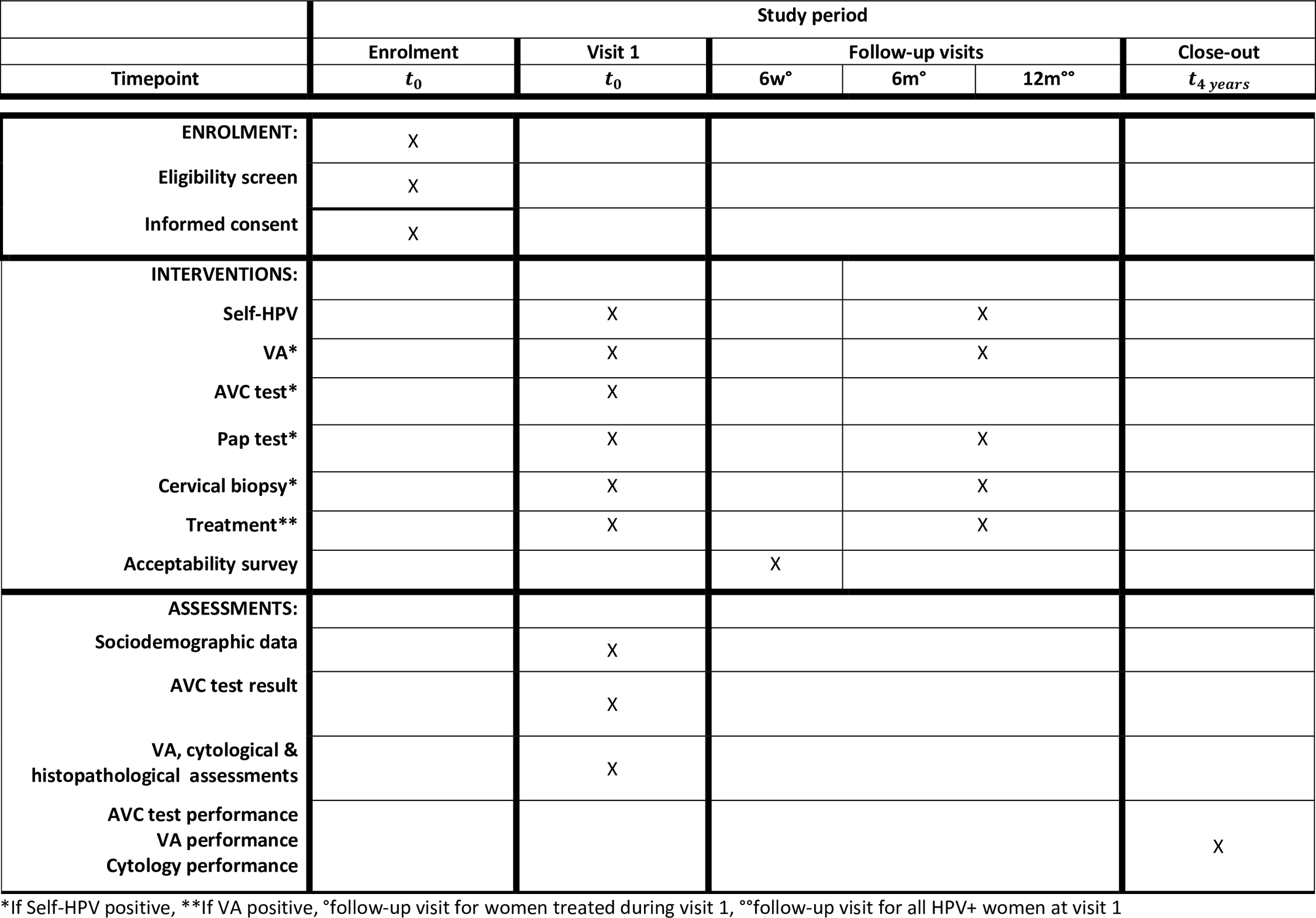

Supplement: S1 Fig — (TIF) [file pone.0260776.s002.tif]
