# Supplementary material for: Study protocol for a two-site clinical trial to validate a smartphone-based artificial intelligence classifier identifying cervical precancer and cancer in HPV-positive women in Cameroon
Source: PLoS One. 2021 Dec 16;16(12):e0260776. doi: 10.1371/journal.pone.0260776 (PMC8675688; doi:10.1371/journal.pone.0260776)
Supplement: S1 File — (PDF) [file pone.0260776.s003.pdf]

Service du pharmacien cantonal
Commission cantonale d'éthique de
la recherche (CCER)
Rue Adrien-Lachenal 8
1207 Genève

N/réf. : BH/fr

Pr Patrick Petignat
Chef du Service de Gynécologie
Département de Gynécologie-Obstétrique
HUG
Bd de la Cluse 30
1205 Genève

Genève, le 6 juillet 2021

Décision de la Commission cantonale d'éthique de la recherche CCER

**Amendement N°4 :**	Plusieurs améliorations nécessitant des modification mineures dans le protocole d'étude.
**Soumis le**	05.07.2021
**Project-ID**	2017-01110
**Titre du projet**	Promoting Comprehensive Cervical Cancer Prevention and Better Women Health in Cameroon
**Investigateur principal**	Pr. Dr. Patrick Petignat
**Promoteur**	Pr. Dr. Patrick Petignat
**Centres**	Pr. Dr. Patrick Petignat, Hôpitaux Universitaires de Genève, Geneva

Procédure de décision

☐ Procédure simplifiée ☒ Procédure présidentielle

Décision

Pr. Dr. Patrick Petignat, Hôpitaux Universitaires de Genève, Geneva

☒ **Autorisation accordée**

Taxes et émoluments : Nihil

Voies de recours

La décision peut faire l'objet d'un recours à la chambre administrative de la Cour de Justice dans un délai de 30 jours dès leur notification (Art 132. loi sur l'organisation judiciaire et loi sur la procédure administrative).

Promoting Comprehensive Cervical Cancer Prevention and Better Women Health in Cameroon

Clinical Study Protocol 30.06.2021

Version 7

**Title**	Promoting comprehensive cervical cancer prevention and better women health in Cameroon
**Study type**	Prospective cohort study
**Study registration**	ClinicalTrials.gov number NCT03757299
**Type of Research Project:**	Research project in which biological material is sampled from humans and/or health-related personal data is collected
**Risk Categorisation:**	Category A
**Project Identifier:**	Not applicable
**Principal investigator:**	Prof. Patrick Petignat<br>Chief of the Gynecology Division<br>Geneva University Hospitals<br>Boulevard de la Cluse 30<br>1205 Geneva<br>Phone: +41 (0)22 37 24 432<br>Email: Patrick.Petignat@hcuge.ch
**Health condition / problem**	Cervical cancer and cervical precancerous lesions
**Project Duration**	September 2018 –September 2024
**Protocol Version and Date:**	Version 7, 30 June 2021
**Amendments to the Protocol:**	N° 4: November 5, 2018<br>N° 5: September 2, 2019<br>N° 6: April 20, 2020

ACCESS TO RESEARCH DOCUMENTS

The current project is not in conflict with any applicable transparency rules.

Table des matières

1. SIGNATURE PAGE 1 4
2. SIGNATURE PAGE 2 5
3. SIGNATURE PAGE 3 6
4. SYNOPSIS (SUMMARY) in English and French 7
5. ABBREVIATIONS..... 12
6. SCHEDULE OF ASSESSMENTS (FLOW CHART AND TIMELINE OF RESEARCH PROJECT) 13
7. ADMINISTRATIVE STRUCTURE..... 15
8. ETHICAL AND REGULATORY ASPECTS 17
8.1 Ethical Conduct of Study 17
8.2 Risk categorization 17
8.3 Ethics Committee (EC), Competent Authorities (CA) and Conflict of interest 17
8.4 Participant Information and Informed Consent 17
8.5 Participant privacy and safety 18
8.6 Early termination of project 18
8.7 Amendments, Changes..... 18
9. INTRODUCTION 20
9.1 Background..... 20
9.2 Rationale for the research project 21
9.3 Risk-Benefit Assessment..... 22
10. OBJECTIVES, ENDPOINTS/ OUTCOMES AND OTHER STUDY VARIABLES 23
10.1 Objectives..... 23
10.1.1 Main objective 23
10.1.2 Secondary objectives 23
10.1.3 Primary endpoint/outcome..... 23
10.1.4 Secondary endpoint/outcomes..... 23
11. PROJECT DESIGN..... 24
11.1 Type of research and general project design 24
11.1.1 Type of research 24
11.1.2 Study design..... 24
12. PROJECT POPULATION..... 27
12.1 Inclusion criteria 27
12.2 Exclusion criteria..... 28
12.3 Criteria for withdrawal / discontinuation of participants 28
13. PROJECT ASSESSMENTS..... 29
13.1 Project flow chart/ table of procedures and assessments..... 29
13.2 Assessments of primary endpoint/ outcome 30

13.3 Assessment of secondary endpoint/ outcomes 30
13.4 Assessment of other study variables 30
13.5 Assessment of safety and reporting 30
13.5.1 Definition of Serious Events (SEs) 30
13.5.2 Assessment and Documentation of SEs 31
13.5.3 Reporting of SAEs, Safety and Protective Measures 31
14. STATISTICAL METHODOLOGY 32
14.1 Determination of Sample Size 32
14.2 Data processing 32
14.3 Planned analysis 33
14.3.1 Datasets to be analyzed 33
14.3.2 Handling of missing data 33
14.3.3 Deviations from the original statistical plan 33
15. DATA AND QUALITY MANAGEMENT 33
15.1 Data handling and record keeping / archiving 33
15.2 Coding and back-up 34
15.3 Confidentiality, Data Protection 34
15.4 Archiving and Destruction 34
15.5 Monitoring and audits/inspections 34
16. PUBLICATION AND DISSEMINATION POLICY 34
16.1 Publication of results 34
16.2 FUNDING AND SUPPORT 35
16.3 INSURANCE 35
17. REFERENCES 36

SYNOPSIS (SUMMARY) IN ENGLISH AND FRENCH

**Project Leader (or Sponsor)**	Professor Patrick Petignat<br>Chief of the Gynaecology Division<br>Geneva University Hospitals<br>Boulevard de la Cluse 30; CH-1205 Geneva Tel: +41 (0)22 37 24 432<br>Email: Patrick.Petignat@hcuge.ch
**Study Title:**	Promoting Comprehensive Cervical Cancer Prevention and Better Women Health in Cameroon
**Protocol Version/Date:**	Version 7<br>30.06.2021
**Study Category with Rationale**	- Risk Category A: study with minimal or no risk for study participants<br>- Research project in which biological material is sampled and/or health-related personal data is collected or project in which biological material and/or health-related personal data are used for further research. Coded data will be used.
**Background and Rationale:**	In sub-Saharan Africa, cervical cancer is the leading cause of cancer death among women because of the difficulty in implementing screening programs. The main obstacles in these countries are poverty, lack of healthcare infrastructures and trained practitioners. With the availability of new technologies, researchers are looking for new strategies adapted to low- and middle-income countries to identify cervical precancerous lesions.<br>Current evidence shows that Human Papilloma Virus (HPV) testing is more effective than cytology (Pap smear) for cervical cancer screening in resource-limited settings. Indeed, the GeneXpert® HPV test offers the opportunity to prevent cervical cancer (CC) in a single visit: rapid detection of high-risk HPV (HPV) infection followed by same day treatment of HPV-positive women during the same visit (screen-and-treat approach).<br>However only a small proportion of HPV-positive women will develop cervical (pre)cancer, making it important to select those to treat. This triage can be achieved by colposcopy, cytology and visual inspection after application of acetic acid (VIA). Though VIA is the triage test recommended by WHO for resource-limited countries, it has not yet been widely assessed in sub-Saharan Africa (SSA). Simplified WHO criteria (ABCD) will be used to improve the VIA sensitivity.<br>Our main objective is to assess the performance of HPV-test followed by VIA and Lugol's iodine VIA/VILI based on smartphone images to detect cervical precancerous lesions in a screen-and-treat strategy.<br>We organized a successful free screening campaign in Cameroon in 2015 that allowed us to identify the expectations of women and their eagerness to benefit from prevention of gynecological cancers and sexually transmitted diseases.

**Objective(s):**	Primary objective<br>- To determine the sensitivity and specificity HPV test followed by VIA/VILI to detect cervical precancerous lesions in a screen-and- treat strategy using histology as gold standard<br>Secondary objectives:<br>- To create a database of cervical images for continuous clinical education and research,<br>- To assess the impact of HPV-positive screening on the level the women's quality of life among women in Cameroon,<br>- To examine the impact of the screening on sexual and reproductive health among women with HPV,<br>- To assess the impact of HPV-positive screening on the level of anxiety among women,<br>- To better understand the accessibility of HPV-self sampling and potential barriers to screening,<br>- To inform women and their families about gynecological pathologies, including cervical cancer, sexually transmitted diseases (including HIV) and vaginal fistula,<br>- To assess women acceptability of self-HPV, thermal ablation and screening process,<br>- To study the HPV infection positivity and HPV clearance, as well as the prevalence of cervical cancer and pre-cancer in our population,<br>- To assess thermal ablation efficacy for the treatment of precancerous lesions,<br>- To offer the possibility to perform HIV test in HIV center in Dschang,<br>- To expand coverage rate and improve access to cervical cancer screening programs with participation of community health workers.<br>- To develop a detection algorithm using cervical images that can identify cervical precancerous lesion<br>- To assess the impact of thermal ablation on fertility and obstetrical outcomes<br>- To develop an Automated VIA Classifier (AVC) that can help identify cervical precancerous lesions based on a 2-minute video of the cervix during VIA and machine learning<br>- To assess women's, the community's and healthcare providers' acceptability of the AVC test<br>- To assess the AVC test performance
**Outcomes:**	Primary Outcome<br>- Sensitivity and specificity of HPV test followed by VIA/VILI to detect cervical precancerous lesions in sub-Saharan Africa using histology as gold standard<br>Secondary outcomes<br>- Provide teaching material for training health care professionals on cervical cancer prevention through VIA/VILI (cervical images database),<br>- Sexual dysfunction score, score of anxiety and method of contraception after screening procedures,<br>- Willingness to participate in the 3T screening program,<br>- Prevalence of HPV infection, cervical pre-cancer and cancer,<br>- HPV clearance measured by self HPV at 6 and 12 months follow up,<br>- Prevalence of CIN2+ disease at the 12-month follow-up,<br>- Acceptability of self-HPV test, thermal ablation and cervical cancer screening procedures,<br>- Women's experiences and attitudes on cervical cancer screening<br>- Proportion of side effects and complications after thermal ablation or LEEP,<br>- VIA test-positivity rate (HPV-positive women);<br>- VIA test-positivity rate at 1- year follow-up of VIA-negative tests<br>- Thermal ablation efficacy<br>- Increase awareness on gynecological pathologies, including cervical

	cancer, sexually transmitted diseases and HIV, vaginal fistula in the community of the study area,<br>- Number of women screened with and without community health care workers.<br>- Specificity and sensitivity of the AVC test, using histology as reference standard<br>- Acceptability of the AVC test
**Study Design**	Prospective cohort study
**Inclusion/Exclusion Criteria**	Inclusion criteria:<br>- Women aged 30-49 years, able to comply with the study protocol<br>Exclusion criteria<br>- Pregnancy<br>- Previous total hysterectomy<br>- Conditions impairing examination of the cervix
**Measurements and Procedures:**	Primary (Flow chart)<br>- After a community-based sensitization campaign, participants will undergo HPV-testing with GeneXpert® machines using self- collected samples (Flocked swabs) transported in a 0.9% sodium chloride (NaCl 0.9%) medium,<br>- HPV-negative women will be reassured and advised to do the next screening 5 years later,<br>- HPV-positive women will undergo the following sequence on the same day: VIA/VILI > Pap smear > Cervical Biopsy (VIA-guided biopsy for VIA-positive participants and random biopsy for VIA- negative participants) > Endocervical Curettage (ECC). All participants with a positive VIA/VILI will be treated with thermal ablation if eligible or Loop Electrosurgical Excision Procedure ((LEEP) for participants ineligible for thermal ablation).<br>- Specimens of Pap smear will be transported in *BD SurePath™* vials (Becton Dickinson Company, Franklin Lakes New Jersey, USA) or *ThinPrep™* (Hologic, Bedford, MA, USA) while those of biopsy, LEEP and ECC will be transported separately in formol (formaldehyde solution) vials.<br>- VIA/VILI will be interpreted on Smartphone images according to ABCD criteria and compared to VIA.<br>- Results of cytological and histological analysis will be disclosed to participants by midwives or obstetrician-gynecologist. Those requiring additional treatment will receive it for free.<br>- Follow-up visits will be organized 6 and 12 months later for women having received a treatment (self HPV GeneXpert® test > VIA/VILI > Pap smear > Guided biopsy + ECC).<br>Follow-up visits will be organized 12 months later for women having a positive screening test but VIA/VILI negative (self HPV GeneXpert® test > VIA/VILI > Pap smear > Guided biopsy + ECC)<br>Secondary<br>- Pictures of the cervix (native, after application of acetic acid (VIA) and lugol's iodine (VILI)) will be taken using a Smartphone (Samsung Galaxy™ S5). In addition to these 3 pictures, a 120 second long video of the cervix (120 images) will be recorded during the VIA. Then a database will be built-up.<br>- During the visit, participants will receive information on gynecological pathologies, including cervical cancer, sexually transmitted diseases and HIV, vaginal fistula<br>- All participants diagnosed with pre-cancer or cancer of the cervix will receive free of charge treatment<br>- Participants treated by thermal ablation and an equivalent number of untreated patients (about 250 in each group) will be followed up by phone or interview for a survey on fertility and obstetrical

	outcomes. Participants from a previous study on cervical screening in Dschang, conducted by our study group, will be contacted as well for this follow-up study (about 50 in each group). Information about obstetrical outcomes may also be retrieved from medical records.
**Number of Participants with Rationale (if no Power Analysis conducted):**	We plan to include 6000 women in the study. Assuming a prevalence of HPV infection of 20%, 1200 participants will be HPV+.<br>- Among the awaited 1200 HPV+ women, 120 will have CIN2+ lesions (assuming a prevalence of CIN2+ of 10% among HPV+ women). Assuming a 20% drop-out rate, 96 cases of CIN2+ will be analyzed to determine the sensitivity of VIA (expected around 60%) with a precision of +/- 9.8%.<br>- Among the awaited 1200 HPV+ women, 1080 will not have CIN2+ lesions. With a drop-out rate of 20%, 864 cases of non-CIN2+ will be analyzed to determine the specificity of VIA (expected around 50%) with a precision of +/- 3.3%.
**Study Duration:**	72 months
**Study Schedule:**	Month Year of First-Participant-In (planned): September 2018<br>Month Year of Last-Participant-In~~Out~~ (planned): September 2024
**Principal Investigator:**	Dr Engelbert Manga<br>Division of Cooperation<br>Ministry of Health<br>Tel: +237 699 922 032<br>Email: engemanga@gmail.com
**Study Centre(s):**	The project will take place in Dschang District Hospital
**Statistical Analysis incl. Power Analysis**	We will determine the sensitivity, specificity, positive and negative predictive values of VIA/VILI to detect CIN2+ among HPV-positive women. Histology will be used as gold standard. All these will be done using a 95% confidence interval.
**GCP Statement:**	This study will be conducted in compliance with the protocol, the current version of the Declaration of Helsinki, the ICH-GCP as well as all national legal and regulatory requirements.

**Promoteur/Sponsor**	Prof. Patrick Petignat<br>Chef du service de Gynécologie<br>Hôpitaux Universitaires de Genève<br>Boulevard de la Cluse 30; CH-1205 Genève<br>Tél: +41 (0)22 37 24 432<br>Email: patrick.petignat@hcuge.ch
**Titre**	Promotion du dépistage du cancer du col de l'utérus et de la santé de la femme au Cameroun
**Version et Date du protocole:**	Version 7<br>30.06.2021
**Catégorie de risque et justification**	- Catégorie A: Risque minimal ou nul pour les participantes<br>- Projet de recherche au cours duquel du matériel biologique est prélevé et/ou des données sanitaires personnelles sont récoltées.<br>- Le codage des données sera systématique.
**Background**	En Afrique sub-saharienne, le cancer du col de l'utérus est la première cause de mortalité par cancer chez la femme du fait de l'absence ou de l'inefficacité des programmes de dépistage. Les principaux obstacles dans ces pays sont : la pauvreté, le déficit d'infrastructures de santé et de personnel formé. Avec l'avènement de nouvelles technologies, la recherche de stratégies de dépistage adaptées aux contextes à ressources limitées a été redynamisée.<br>Il a récemment été établi que le test HPV (Human Papilloma Virus) est plus efficace que la cytologie (Frottis cervical) pour le dépistage du cancer du col (CC) dans les pays à ressources limitées. En effet, le GeneXpert® HVP permet de prévenir le cancer du col en une seule visite: test HPV rapide et traitement immédiat (approche Dépister-et-traiter).<br>Toutefois, seule une petite proportion des femmes HPV-positives auront un pré cancer du col utérin, ce qui impose une sélection de femmes HPV-positives à traiter. Ce triage peut être fait de trois façons: la colposcopie, la cytologie, et l'inspection visuelle après application d'acide acétique (IVA).Bien que l'IVA soit la méthode de triage recommandée par l'Organisation Mondiale de la Santé (OMS) pour les pays à ressources limitées, elle n'a pas encore été évaluée à grande échelle en Afrique sub-saharienne (ASS). Des critères de l'OMS simplifiés (ABCD) seront utilisés afin d'améliorer la sensibilité de l'IVA.<br>L'objectif principal de cette étude est d'évaluer la performance du test HPV suivi de l'inspection visuelle après application d'acide acétique et de lugol (IVA/IVL) à partir de photographies par smartphone pour la détection des lésions précancéreuses du col de l'utérus dans une approche « dépister et traiter » au Cameroun.<br>Une campagne de dépistage a été organisée avec succès au Cameroun en 2015, ce qui nous a permis d'identifier les attentes des femmes et leur motivation à bénéficier d'une prévention de cancers gynécologiques et maladies sexuellement transmissibles.
**Objectifs:**	<u>Objectif principal</u><br>- Déterminer la sensibilité et la spécificité du test HPV suivi de l'IVA/IVL pour détecter les lésions précancéreuses du col utérin en se référant à l'histologie comme étalon d'or<br><u>Objectifs secondaires :</u><br>- Créer une base de données d'images cervicales pour soutenir la formation médicale continue et la recherche ;<br>- Déterminer l'impact d'un dépistage HPV-positif sur le niveau de qualité de vie des femmes au Cameroun ;

	- Evaluer l'impact du dépistage sur la santé sexuelle et reproductive parmi les femmes HPV-positives ;<br>- Evaluer l'impact d'un dépistage HPV-positif sur le niveau d'anxiété des femmes ;<br>- Mieux comprendre l'accessibilité de l'autoprélèvement du HPV et les potentielles barrières au dépistage ;<br>- Informer les femmes sur les pathologies gynécologiques (CC, maladies sexuellement transmissibles (y compris le VIH), fistule obstétricale) ;<br>- Evaluer l'acceptabilité de l'auto-dépistage HPV, la thermal ablation et le processus de dépistage chez les femmes ;<br>- Déterminer le taux de positivité et de clearance des infections à HPV, la prévalence de pré-cancers et cancers cervicaux dans notre population d'étude ;<br>- Evaluer l'efficacité de la thermal ablation pour le traitement de lésions précancéreuses ;<br>- Offrir la possibilité d'effectuer un test VIH dans un centre VIH à Dschang ;<br>- Etendre le taux de couverture de dépistage et améliorer l'accès au programme de dépistage du cancer cervical à l'aide de la participation d'agents de santé communautaires.<br>- Evaluer l'impact de la thermoablation sur la fertilité et les issues obstétricales<br>- Développer un algorithme d'intelligence artificielle permettant d'identifier les lésions précancéreuses<br>- Développer un outil de classification automatique (AVC) pour l'IVA permettant d'aider à identifier les lésions précancéreuses à partir d'une vidéo de 2 minutes du col de l'utérus et d'intelligence artificielle<br>- Evaluer l'acceptabilité du test AVC par les femmes, la communauté et le personnel de santé<br>- Evaluer la performance du test AVC
**Issues**	<u>Issue primaire</u><br>- Sensibilité et spécificité du test HPV suivi de l'IVA/IVL pour détecter les lésions précancéreuses du col utérin en se référant à l'histologie comme étalon d'or<br><u>Issues secondaires</u><br>- Score de dysfonction sexuelle, score d'anxiété et méthodes de contraception utilisées après le dépistage ;<br>- Volonté de participer au programme de dépistage 3T ;<br>- Prévalence de l'infection à HPV, des lésions précancéreuses et cancéreuses du col de l'utérus chez la femme Camerounaise ;<br>- Clairance des infections à HPV mesurée par autoprélèvement aux visites de suivi de 6 et 12 mois ;<br>- Prévalence de lésions précancéreuses CIN2+ à la visite de suivi de 12 mois ;<br>- Acceptabilité des procédures du test d'auto prélèvement, de la thermal ablation et du dépistage du cancer du col de l'utérus ;<br>- Proportion d'effets indésirables et complications suite aux traitements par thermal ablation et LEEP ;<br>- Taux de positivité de la VIA (HPV-positive women);<br>- Taux de positivité de la VIA qui après 1 an de suivi chez les femmes VIA-négatives<br>- Efficacité de la thermoablation<br>- Augmentation des connaissances sur les pathologies gynécologiques (CC, maladies sexuellement transmissibles (y compris le VIH), fistule obstétricale) dans la région de l'étude ;<br>- Etablissement d'une base de données d'images cervicales après IVA/IVL pour la formation des prestataires de santé sur le

	dépistage du cancer du col;<br>- Nombre de femmes dépistées par l'intégration d'agents de santé communautaire ou non pour le recrutement de femmes.<br>- Base de données d'images cervicales pour la formation des prestataires de santé;<br>- Spécificité et sensibilité du test AVC en utilisant comme référence le résultat d'histologie<br>- Acceptabilité du test AVC
**Type d'étude**	Etude de cohorte prospective.
**Critères d'inclusion et d'exclusion**	Critères d'inclusion:<br>- Femmes âgées de 30-49 ans capables d'observer le protocole d'étude.<br>Critères d'exclusion<br>- Grossesse<br>- Hystérectomie totale<br>- Pathologies empêchant de visualiser le col utérin
**Measures et Procédures:**	Objectif primaire (Flow chart)<br>- Après une campagne de sensibilisation communautaire, les participantes feront un auto-prélèvement vaginal avec des bâtonnets floqués transportés dans un milieu de Chlorure de Sodium à 0,9% en vue d'un test HPV rapide (GeneXpert®).<br>- Les participantes HPV-négatives seront rassurées et conseillées en vue d'un prochain test HPV cinq ans plus tard.<br>- Les participantes HPV-positives suivront la séquence ci-après le même jour: IVA/IVL > Frottis cervical > Biopsie cervicale (guidée par l'IVA pour les participantes IVA-positives et aléatoire pour les patientes IVA-négatives) > Curetage endocervical (CEC). Toutes les participantes IVA/IVL-positives seront traitées parthermal coagulation ou par conisation à l'anse diathermique selon l'éligibilité.<br>- L'IVA/IVL seront interprétés sur la base de photographies smartphones selon les critères ABCD<br>- Les spécimens de frottis cervical seront transportés dans des flacons *BD SurePath*™ (Becton Dickinson Company, Franklin Lakes New Jersey, USA) ou *ThinPrep*™ (Hologic, Bedford, MA, USA) et ceux des biopsies, de CEC et de conisation seront transportés séparément dans du formol.<br>- Les résultats des analyses cytologiques et histologiques seront expliqués aux participantes par une sage-femme ou un gynécologue-obstétricien-ne. Celles ayant des indications à un traitement supplémentaire le recevront gratuitement.<br>- Des visites de suivi seront organisées à 6 et 12 mois pour les femmes qui ont été traitées (HPV GeneXpert® test par autoprélèvement > IVA/IVL > frottis cervical > biopsie guidée + CEC).<br><br>Objectifs secondaires<br>- Des photographies du col (natif et après application d'acide acétique et du lugol) seront réalisées avec un smartphone (Samsung Galaxy™ S5). En plus des 3 photographies, une vidéo de 2 minutes (120 images) sera prise lors de l'IVA. Elles seront utilisées pour constituer une base de données.<br>- Au cours de la première visite, les participantes recevront des informations sur les pathologies gynécologiques (cancer du col de l'utérus, fistules obstétricales et maladies sexuellement transmissibles).<br>- Tous les cancers et pré-cancers du col utérin dépistés seront traités gratuitement.

	- Les participantes traitées par thermoablation et un groupe contrôle de taille équivalente (environ 250 femmes dans chaque groupe) seront suivies par téléphone ou entretien pour évaluer les issues obstétricales et sur la fertilité. Les participantes d'une précédente étude sur le dépistage du cancer du col menée par notre groupe de recherche à Dschang, seront également contactées pour cette étude de suivi (environ 50 dans chaque groupe). Des données sur les issues obstétricales pourront également être obtenues à partir des dossiers médicaux des participantes
**Nombre de participants et justificatif:**	Nous inclurons 6000 participantes. Avec une prévalence d'infection à HPV supposée de 20% nous nous attendons à 1200 participants HPV+.<br>Parmi ces 1200 participantes HPV+, 120 auront des lésions intra épithéliales de haut grade (CIN2+) en considérant une prévalence de 10% de CIN2+ chez les HPV+. En supposant un taux de perdues de vue de 20%, 96 cas de CIN2+ seront analysés pour déterminer la sensibilité de l'IVA (estimée à environ 60%) avec une précision de +/- 9,8%.<br>Parmi ces 1200 participantes HPV+, 1080 n'auront pas de lésions CIN2+. Si le taux de perdues de vue est de 20%, 864 cas sans lésions CIN2+ seront analysés pour déterminer la spécificité de la VIA (estimée à environ 50 %) avec une précision de +/-3,3%.
**Durée de l'étude:**	72 mois
**Planification de l'étude:**	Recrutement de la première patiente : Septembre 2018<br>Recrutement de la dernière patiente : Septembre 2024
**Investigateur principal :**	Dr Engelbert Manga<br>Division de la coopération<br>Ministère de la santé publique/Cameroun<br>Tel: +237 699 922 032<br>Email: engemanga@gmail.com
**Sites de l'étude:**	Hopital de District Dschang
**Analysis statistique**	Nous calculerons la sensibilité, la spécificité et les valeurs prédictives positives et négatives de l'IVA pour détecter les lésions CIN2+ chez les femmes HPV+. L'histologie sera servira d'étalon d'or et l'intervalle de confiance sera de 95%.
**Bonne pratique clinique:**	Nous conduirons cette étude dans le strict respect du protocole et en conformité avec la version en vigueur de la déclaration d'Helsinki et la conférence internationale d'harmonisation des bonnes pratiques cliniques en recherche. Nous observerons aussi toutes les lois et règlements nationaux en matière de recherche.

ABBREVIATIONS

**ABCD**	A=Acetowitheness, B=Bleeding, C=Coloring, D=Diameter
**AVC**	Automated VIA Classifier
**CA**	Competent Authorities
**CC**	Cervical cancer
**CEC**	Competent Ethics Committee
**CIN**	Cervical Intra Epithelial Neoplasia
**CIN1**	Cervical Intra Epithelial Neoplasia grade 1
**CIN2+**	Cervical Intra Epithelial Neoplasia grade 2 or above
**dr-WET**	HPV test performed by a physician or nurses immediately immersed in ThinPrep® PreservCyt® Solution
**EPFL**	Ecole Polytechnique Fédérale de Lausanne
**FIGO**	International Federation of Gynecology and Obstetrics
**GCP**	Good Clinical Practice
**HIV**	Human Immunodeficiency Virus
**HPV**	Human Papillomavirus
**HUG**	Hôpitaux Universitaires de Genève
**ICF**	Informed Consent Form
**ml**	Milliliters
**NGO**	Non-governmental organization
**PCR**	Polymerase Chain Reaction
**S-DRY**	vaginal self-sampling using dry swabs later immersed in NaCl 0.9% medium
**SE**	Serious Event
**STD**	Sexually transmitted diseases
**VIA**	Visual Inspection with Acetic Acid
**VILI**	Visual inspection with Lugol´s iodine
**WHO**	World Health Organization

1. SCHEDULE OF ASSESSMENTS (FLOW CHART AND TIMELINE OF RESEARCH PROJECT)

	08/ 17	09/17	10/17 - 08/18	09-12/18	01-02/18	03-08/19	09-12/19	01-08/20	09/20-09/23	09/23-09/24	10-12/24	01-02/25
Seeking approval by Ethic Commissions :												
CE Geneva												
CE Cameroon												
Community sensitization (campaigns, media, CHWs)												
Identification and training of Cameroonian health providers for study purposes												
Cervical cancer screen-and-treat campaign												
Follow-up visits at 6 months												
Follow-up visits at 12 months												
Disclosure of cytology and histology results												
Data analysis and publication of results												

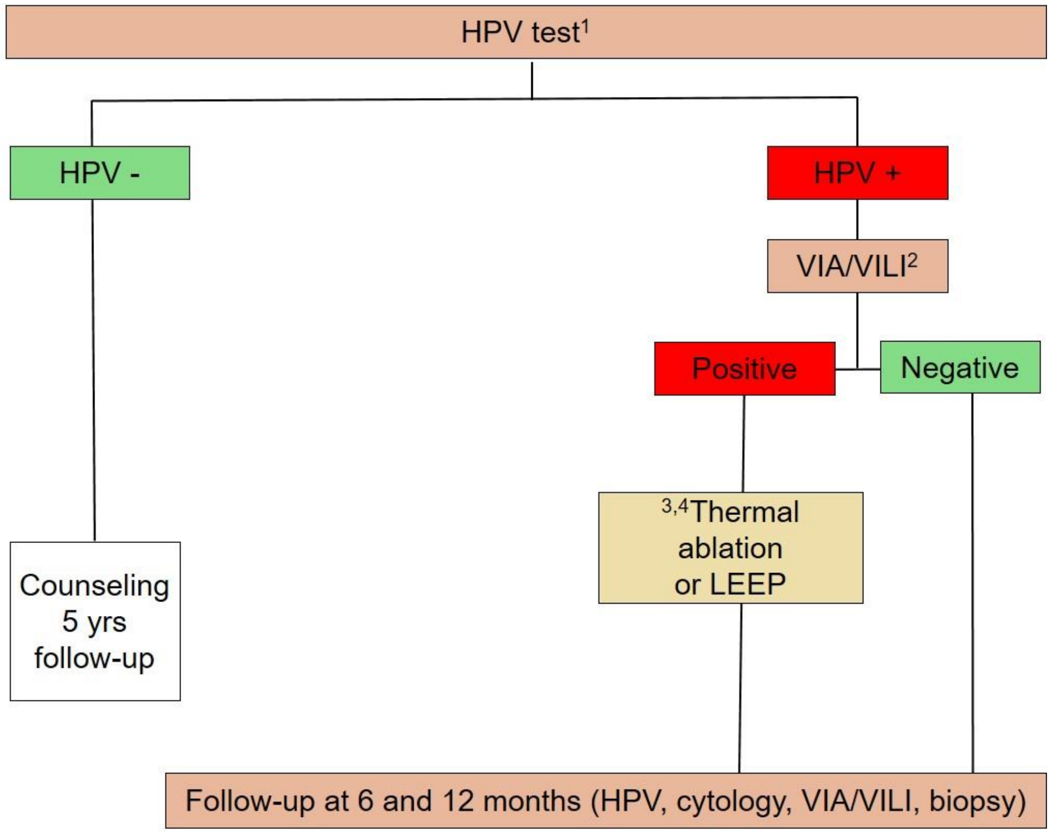

[1]Use of Xpert test; [2]Inspection for eligibility for thermal ablation using acetic acid and Lugol; [3]If not eligible for cold coagulation, refer LEEP; [4]If suspicious for cancer refer to tertiary center; HPV: Human papillomavirus; LEEP: Loop electrosurgical excision procedure; VIA: Visual inspection with acetic acid; VILI: Visual inspection with Lugol iodine

Figure 1. Flow chart of the study

[1]Use of GeneXpert® test; [2]If suspicious of cancer refer to tertiary center; [3]All HPV positive women will have as quality control a cytology and guided biopsy (or random biopsy if VIA/VILI normal); a recall will be done if abnormal cytology/histology in untreated women. [4]LEEP (Loop Electrosurgical Excision Procedure) in case of ineligibility to thermal ablation.

2. ADMINISTRATIVE STRUCTURE

**Sponsor/Promoter**	- Prof. Patrick Petignat<br>Chief of the Gynecology Division Geneva University Hospitals Boulevard de la Cluse 30<br>CH-1205 Geneva<br>Tel: +41 (0)22 37 24 432<br>Email: Patrick.Petignat@hcuge.ch
**Principal investigator**	- Dr Engelbert Manga, MD, MPH.<br>Head of the International Cooperation Unit Ministry of Health Cameroon.<br>Tel: +237 699 92 20 32 Email:<br>Email: engemanga@gmail.com
**Project sites and responsible researchers**	- Dr Bruno Kenfack, MD. Head of the Gynaeco- Obstetric Unit of the Dschang District Hospital (Dschang - Cameroon). Tel: +237 677 779 224 Email Email: brkenfack@yahoo.fr<br>- Pr Jean-Philippe Thiran, Director of the Signal Processing Laboratory (LTS5), at the Swiss Federal Institute of Technology Lausanne (EPFL)
**Co-investigators in Cameroon**	- Pr Pierre-Marie Tebeu, MD, MPH, Professor. Head of Gynaeco-Obstetric unit of the Yaounde University Teaching Hospital (Cameroon).Tel: +237 677006<br>718 Email: pmtebeu@yahoo.fr<br>- Dr Jovanny Tsuala Fouogue MD. Gynecology Division, Mbouda District Hospital, PO Box: 23 Tel<br>+237 233 306 106. University Hospitals of Geneva, Switzerland. Boulevard de la Cluse 30, 1205 Genève.<br>Email fotsujo@outlook.com<br>- Pr Zacharie Sando, MD, MPH, Professor. Head of the Pathology Unit of the Yaounde Gynaeco- Obstetric Hospital. Cameroon Tel: +237 677 78741 Email: zandozac@yahoo.fr
**Biostatistician**	- Assistance for data analysis may be requested at a later stage to the CRC (Centre de Recherche Clinique), Geneva, Switzerland
**Co-investigator in Geneva**	- PD, Dr Pierre Vassilakos, Geneva Foundation for Medical Education and Research, Geneva, Switzerland. Email: pierrevassilakos@bluewin.ch<br>- PhD candidate, Jessica Sormani, University Hospitals of Geneva, Switzerland. Tel: +41 (0)22 37 24 356. Email: jessica.sormani@hcuge.ch<br>- Research assistant, Dr Ania Wisniak, University Hospitals of Geneva, Switzerland. Email: ania.wisniak@hcuge.ch<br>- PhD Candidate, Dr. Sophie Lemoupa, University Hospitals of Geneva, Switzerland. Email: sophie.lemoupa@hcuge.ch<br>Dr Rosa Catarino, University Hospitals of Geneva, Switzerland. Email: rosapintocatarino@gmail.com
**Co-investigator in Lausanne**	- Prof, Dr Jean-Philippe Thiran, Signal Processing Laboratory LTS5, Swiss Federal Institute of Technology Lausanne, Switzerland.<br>EPFL-STI-IEL-LTS5, Station 11, 1015 Lausanne.<br>Email: jean-philippe.thiran@epfl.ch<br>- PhD candidate, Magali Cattin, Signal Processing Laboratory LTS5, Swiss Federal Institute of Technology Lausanne,

Switzerland.
EPFL-STI-IEL-LTS5, Station 11, 1015 Lausanne.
Email: magali.cattin@epfl.ch

- PhD candidate, Roser Viñals Terres, Signal Processing Laboratory LTS5, Swiss Federal Institute of Technology Lausanne, Switzerland.
  EPFL-STI-IEL-LTS5, Station 11, 1015 Lausanne.
  Email: roser.vinalsterres@epfl.ch
- Deputy Director, Dr Solomzi Makohliso, EssentialTech Centre, Swiss Federal Institute of Technology Lausanne, Switzerland.
  Station 10, CM 1 368, 1015 Lausanne.
  Email: solomzi.makohliso@epfl.ch

Laboratory

The laboratory analyses will be carried out :

- HPV tests will be carried out by the GeneXpert® machine (Cepheid. 904 Caribbean Drive. Sunnyvale CA 94089 USA) on the study site.
- Cytological and Histological analysis will be carried out in Yaounde and in Geneva. Rue Gabrielle- Perret Gentil 4, 1211 Genève 14

3. ETHICAL AND REGULATORY ASPECTS

3.1 Ethical Conduct of Study

The research project will be carried out in accordance to the research plan and with principles enunciated in the current version of the Declaration of Helsinki (DoH), the Essentials of Good Epidemiological Practice Issued by Public Health Schweiz (EGEP), The Swiss Law and Swiss regulatory authority´s requirements as applicable.

The study will be carried out in a setting where there is no structured screening program. WHO recommends HPV testing followed by VIA/VILI assessment as an option. These screening procedures are highly accepted and well tolerated by women. The side effects are usually mild and with no consequence on women's health. Local doctors, in accordance with the recommendations of FIGO and WHO, will manage women with cervical dysplasia or cancer.

3.1. Risk categorization

The study's risk category is A. This decision is based on the fact that the planned measures for sampling biological material or collecting personal data entail only minimal risks and burdens.

3.2. Ethics Committee (EC), Competent Authorities (CA) and Conflict of interest

The responsible investigator will make sure that approval is obtained from the three following institutions before beginning three study: the Geneva Cantonal Ethics Committee on Human Research, the Cameroon National Ethics Committee and the Cameroonian Ministry of Health.

The regular end, premature end or interruption of the research project is reported to the EC within 90 days upon completion of the project.

The principal investigator and co-investigators have no conflict of interest for the present study protocol.

3.3. Participant Information and Informed Consent

The investigators will explain to each participant the nature of the study, its purpose, the procedures involved, the expected duration, the potential risks, benefits and discomfort it may entail. Each participant will be informed that the participation in the study is voluntary and that she may withdraw from the study at any time and that withdrawal of consent will not affect his/her subsequent medical assistance and treatment.

The participant must be informed that her medical records may be examined by authorized individuals other than their treating physician.

All participants for the study will be provided a participant information sheet and a consent form describing the study and providing sufficient information for the participant to make an informed decision about their participation in the study. The participant will be given about 3 hours to decide whether to participate or not, which will allow a treatment within the same day.

The patient information sheet and the consent form will be submitted to the CEC and to the competent authority to be reviewed and approved. The formal consent of a participant, using the approved consent form, must be obtained before the participant is submitted to any study procedure.

The participant should read and consider the statement before signing and dating the informed consent form, and should be given a copy of the signed document. The consent form must also be signed and dated by the investigator (or their designee) and it will be retained as part of the study records.

3.4. Participant privacy and safety

The investigator affirms and upholds the principle of the participants' right to dignity, privacy and health and that the project team shall comply with applicable privacy laws. Especially, anonymity of the participants shall be guaranteed when presenting the data at scientific meetings or publishing them in scientific journals.

Individual subject medical information obtained as a result of this study is considered confidential and disclosure to third parties is prohibited. Subject confidentiality will be further ensured by utilizing subject identification code numbers to correspond to treatment data in the computer files. The primary

investigator and their research assistants will store the participant identification list. Only they will have access to the participant's list and the coded database.

For data verification purposes, authorized representatives of the Sponsor (-Investigator), a competent authority or an ethics committee may require direct access to parts of the medical records relevant to the study, including participants' medical history.

3.5. Early termination of project

The Sponsor-Investigator may terminate the study prematurely according to certain circumstances, for example:

- ethical concerns;
- insufficient participant recruitment;
- when the safety of the participants is doubtful or at risk, respectively;
- alterations in accepted clinical practice that make the continuation of a clinical trial unwise.

3.6. Amendments, Changes

Substantial amendments are only implemented after approval of the CEC and CA respectively.

Under emergency circumstances, deviations from the protocol to protect the rights, safety and well-being of human subjects may proceed without prior approval of the sponsor and the CEC/CA. Such deviations shall be documented and reported to the sponsor and the CEC/CA as soon as possible.

All Non-substantial amendments are communicated to the CA as soon as possible if applicable and to the CEC within the Annual Safety Report (ASR).

4. INTRODUCTION

4.1 Background

In sub-Saharan Africa, cervical cancer (CC) remains the leading cause of cancer death among women, accounting for nearly a quarter-million deaths per year [1], because of the difficulty in implementing a screening program. The main obstacles in these countries are poverty, lack of healthcare infrastructures and trained practitioners. Moreover cervical cancers are responsible for complications such as infections and uncontrolled vaginal loss (vaginal fistula).

Recent development of tests for high-risk human papillomavirus (HPV) infection has created an important change in our understanding of CC screening. Overwhelming evidence from several randomized trials has shown that HPV-based screening is more effective than cytology (Pap smear) in preventing CC, with an improved sensitivity and negative predictive value that allow less frequent screening [2, 3].

At the moment, several developing countries, following the recommendations of the World Health Organization (WHO), are evaluating the use of HPV testing as a primary screening tool [4]. A recent study has demonstrated that in medium and low-resource settings, HPV testing was associated with decreased CC-related mortality [5, 6]. Furthermore, HPV testing gives the possibility to perform the collection of the sample by women themselves (Self-HPV), which is considered as accurate as physician-sampling, supporting that Self-HPV is a feasible alternative method for primary CC screening in medium and low-resource settings [4].

A single-visit approach has the potential to increase program effectiveness markedly given the high rates of loss to follow-up that are common in the developing world due to the difficulties that women and their families have in obtaining services (related to transport, clinic hours, costs, child care needs and other barriers). There is now growing evidence supporting that a single-visit approach, by incorporating the diagnostic procedure followed by an immediate treatment is safe, acceptable and cost-effective [4].

Recently, new laboratory-independent HPV test have been made available, providing immediate results and making it possible to screen and treat women on the same day. This aspect is particularly important as the inability to follow through with the necessary treatment for screen-positive women is a major cause of low program impact in medium and low-resource settings. One of this rapid, point-of-care testing is the GeneXpert® HPV assay (Cepheid, Sunnyvale, CA, USA), a non-batch and qualitative real-time PCR assay for the detection of hrHPV DNA. The assay is formatted in a single-use test cartridge and a single test can be completed in 1 hour.

Until now the Xpert HPV test was evaluated with specimens previously collected into ThinPrep® (Hologic) vials containing a methanol-water solution (PreservCyt® transport medium) [7-9]. This approach may be impractical and unavailable, because of flammability, toxicity and cost, which make it hardly applicable in low resource settings.

In a previous study on Self-HPV sampling we found that the test results obtained from swabs transported in a dry state were as accurate as those obtained with swabs shipped in a wet transport medium, in terms of quality of results [10]. Moreover, a pilot study conducted in Geneva in 2015 showed that self-HPV using dry swabs later immersed in NaCl medium (S- DRY) seems to be an equivalent strategy to the cervical collection with the cervix brush immediately immersed in PreservCyt® (Dr-WET).

The possibility of using self-obtained specimens stored at ambient temperature without a transport media would clearly enhance and simplify the utility of Self-HPV. Moreover, it reduces the costs of the method (Preservcyt® is about 5 US dollars per sample and NaCl 0.9% would be 2 cents per sample) which might be attractive for a point-of-care strategy.

Though treating all HPV-positive women may lead to overtreatment (treating women without precancerous lesions) it is recommended by WHO for countries with very limited resources. WHO recommendations for those with little more resources include a triage of HPV-positive women. This triage (that reduces overtreatment rate) is done with VIA, colposcopy and cytology. Among these three tests VIA has been used for several years in low- and middle-income countries; the appearance of aceto-white areas after the application of a 3-5% acetic acid solution helps to define the pathological areas of the cervix. Nevertheless the feasibility/efficacy of VIA as a triaging test has not yet been widely assessed in sub-Saharan Africa. Besides, VIA is a highly subjective procedure dependent on the health care provider's experience, with diagnostic accuracy varying according to the setting [11]–[14]. The reported sensitivities for detecting CIN2+ varies between 25 and 80% between studies [11], [15], [16].

Continuous clinical education is necessary to maintain a high sensitivity of VIA examination. Thus, a large database with different images of the cervix (native image, VIA video, VILI image with the respective histopathological result), would support this pedagogy, allowing healthcare providers to train and improve their capacity of identifying and locating potential cervical lesions.

In addition to further training of healthcare providers, computer-aided diagnosis tools such as the Automated VIA Classifier (AVC) tests could assist the medical staff in their diagnostic decision. So far, several techniques for AVC tests have been investigated [17], most of them relying on static cervical images and showing various performances [18]–[24]. Such tools have the potential to allow large-scale screening and potentially reduce cervical cancer morbidity and mortality.

4.2 Rationale for the research project

Women Health is a priority in Cameroon.

The Ministry of Health showed its interest in the promotion of Women Health, through different documents : Vision 2035, Document de Stratégie pour la Croissance et l'Emploi, La Stratégie Sectorielle de Santé, la Politique Nationale de la Santé de la Reproduction, le Plan Stratégique National de la Santé de la Reproduction, le Plan CARMMA. The government has put gynecological cancers, sexually transmitted diseases and obstetrical fistulas at the top of its agenda.

A free screening campaign was organized in July 2015 in the district of Dschang, Cameroon, in partnership with the Geneva University Hospitals. These campaign was very successful and allowed us to identify the expectations of women and their eagerness to benefit from prevention for gynecological cancers and from information on cancer.

In the Dschang health district, there are about 101,385 inhabitants, including 25,000 women between 30 and 49 years old. HIV prevalence is these two health districts is 4.09% and fistulas account for 1/1000 women.

So the aim of this project is the information, screening and treatment of cervical cancer. Through this program, women will have easier access to exams to screen vaginal fistula and HIV in the health district of Dschang, Cameroon.

For the sustainability of such a screening program in this developing country, the economical aspect has to be taken into account. If the screening strategy including the S-DRY approach with the Xpert

HPV assay flowed by VIA/VILI is efficient then, screening would be faster and would require less trained personal. Moreover, if is feasible, it will help the implementation of a cost-effective screening strategy in developing countries, by overcoming material and human barriers and minimizing need for repeat visits, thus preventing loss to follow-up.

4.3 Risk-Benefit Assessment

The study participants will benefit from a free screening for cervical cancer with HPV test. HPV- positive women will undergo, Pap smear, ECC, VIA/VILI and biopsy. Those with positive VIA will immediately be treated by thermal ablation or LEEP (if not eligible to thermal ablation). All cancer and pre-cancer detected will have free of charge management.

Voluntary women will be addressed to the HIV department for screening and free treatment. Patients with fistulas will be transferred to the University Teaching Hospital of Yaoundé and will be freely treated (collaboration between Pr Pierre-Maire Tebeu and the Swiss NGO "fistula- group" lead by Dr Charles-Henri Rochat, Geneva).

We expect that our data will determine how a CC-screening program might be established in a sustainable prevention strategy. The data will also determine the level of support, which will be needed to maintain high-quality CC prevention services in equipment and provider skills. In addition, the developed AVC tool will assist medical staff in their assessment of VIA and reduce the subjectivity of the process.

One possible risk is the unauthorized data access or unwanted identification of project participants. Another risk though very low is the occurrence of side effects following biopsy and thermal coagulation (light pelvic pain, light vaginal bleeding or watery discharge).

5. OBJECTIVES, ENDPOINTS/ OUTCOMES AND OTHER STUDY VARIABLES

5.1 Objectives

5.1.1 Main objective

- To determine the sensitivity and specificity of VIA/VILI to detect CIN2+ among HPV-positive women, using histology as gold standard

5.1.2 Secondary objectives:

- To create a database of cervical images for continuous clinical education
- To assess the impact of HPV-positive screening on the level the women's quality of life among women in Cameroon
- To examine the impact of the screening on sexual and reproductive health among women with HPV.
- To assess the impact of HPV-positive screening on the level of anxiety among women.
- To better understand the accessibility of HPV-self sampling and potential barriers to screening
- To inform women and their families about gynecological pathologies, including cervical cancer, sexually transmitted diseases (including HIV) and vaginal fistula
- To assess women acceptability of self-HPV, thermal ablation and screening process
- To study the HPV infection positivity and clearance, as well as prevalence of cervical cancer and pre-cancer
- To assess thermal ablation efficacy for the treatment of precancerous lesions
- To offer the possibility to perform HIV test in HIV center in Dschang
- To expand coverage and improve access to cervical cancer screening programs with participation of community health workers.
- To assess the impact of thermal ablation on fertility and obstetrical outcomes
- To develop a detection algorithm using cervical images that can identify cervical precancerous lesion
- To develop an Automated VIA Classifier (AVC) that can help identify cervical precancerous lesions based on a 2-minute video of the cervix during VIA and machine learning
- To assess women's, the community's and healthcare providers' acceptability of the AVC test
  To assess the AVC test performance.

5.2 Endpoints/outcomes

5.2.1 Primary endpoint/outcome

- Specificity and sensitivity of HPV test followed by VIA/VILI to detect CIN2+, using histology as Gold Standard

5.2.2 Secondary endpoint/outcomes

- Provide teaching material for professional training on cervical cancer prevention through VIA/VILI (cervical images database)
- Sexual dysfunction score, score of anxiety and method of contraception after screening procedures
- Willingness to participate in the 3T screening program;
- Prevalence of HPV, cervical pre-cancer and cancer among Cameroonian women
- HPV clearance at 6 and 12 months follow up
- Prevalence of CIN2+ disease at the 12-month follow-up
- Acceptability of self-HPV test, thermal ablation and cervical cancer screening procedures
- Women's experiences and attitudes on cervical cancer screening
- Proportion of side effects and complications after thermal ablation or LEEP
- VIA test-positive rate (HPV-positive women);
- VIA test-positive rate after 1- year follow-up of VIA-negative tests
- Thermal ablation efficacy
- Increase awareness on gynecological pathologies, including cervical cancer, sexually transmitted diseases and HIV, vaginal fistula in the community of the study area
- Number of women screened with and without the participation of community health care workers.
- Specificity and sensitivity of the AVC test, using histology as the reference standard
- Acceptability of the AVC test

The demand for programs to control CC in low-resource settings is strong. Lessons that we have learned from our previous experience, combined with results of recent researches have put our group in an excellent position to identify innovative and cost-effective strategies for reducing CC morbidity and mortality in developing countries. The obtained data will determine the level of support, which will be needed to maintain high-quality CC prevention services in equipment and provider skills. We expect also that our data will also determine how a CC- screening program might be established in a sustainable prevention strategy.

6. PROJECT DESIGN

6.1 Type of research and general project design

6.1.1 Type of research

Research project in which biological material is sampled and/or health-related personal data is collected and will be used for further research including the development of an algorithm for VIA classification. Coded data will be used.

6.1.2 Study design

Single-center prospective and longitudinal (cohort) study

Procedures

The management will be as follows:

<u>First visit</u>

- Information about the study and written informed consent
- Information about cervical cancer, STDs (including HIV) and fistula will be given to women;
- Socio-demographic data, acceptability of the self-test and data on women's quality of life will be collected according to validated questionnaire;
- HPV tests will be carried out on self-collected vaginal (flocked swabs transported in a 0.9% sodium chloride) medium samples using the GeneXpert machine. The results will be available after one hour.
- HPV-negative women will be reassured and advised to do the next screening 5 years later.
- HPV-positive women will undergo the following sequence on the same day: VIA/VILI > Pap smear

> Cervical Biopsy (VIA-guided biopsy for VIA-positive participants and random biopsy for VIA-negative participants) > Endocervical Curettage (ECC). These tests will be done by the following experienced physicians.
- Pictures of the cervix during VIA/VILI (native image, video during acetic acid and image after lugol's iodine) will be stored on a digital device.
- VIA/VILI will be interpreted on smartphone pictures according to the ABCD criteria (relaxed IARC criteria).
- All participants with a positive VIA/VILI will be treated with thermal ablation if eligible or Loop Electrosurgical Excision Procedure (LEEP for participants ineligible to thermal ablation).
- Specimens of Pap smear will be transported in BD SurePath™ vials (Becton Dickinson Company, Franklin Lakes New Jersey, USA) while those of biopsy, LEEP and ECC will be transported separately in formol (formaldehyde solution) vials.
- Cytological and histological analyzes will be conducted both in Yaoundé and in Geneva.

Visit to inform results
- Results of cytological and histological analyses will be explained to participants by a gynecologist-obstetrician (co-investigator). Participant who tested positive for HPV but have normal results for the other exams (VIA/VILI, Pap smear, Cervical biopsy and ECC) will be given an appointment for follow-up at 12 months. Participants with abnormal results will receive comprehensive treatment (thermal ablation or LEEP for CIN2+ and appropriate management for invasive cancers).

Post treatment Visit (follow up at 6 and 12 months)
- Treated patients will be seen at 6 and 12 months post-treatment visits, where they will perform self-HPV GeneXpert® test followed by VIA/VILI, Pap smear, Cervical Biopsy and ECC.
- Participants who will become pregnant during the study shall be counselled on risks excluded from the study and offered focalized antenatal and post-partum care.
- Data on women's quality of life and methods of contraception after screening procedures will be collected according to validated questionnaire;
- Participants treated by thermal ablation and an equivalent number of untreated patients (about 250 in each group) will be followed up by phone or interview for a survey on fertility outcomes. Participants from a previous study on cervical screening in Dschang, conducted by our study group, will be contacted as well for this follow-up study (about 50 in each group). Information about obstetrical outcomes may also be retrieved from medical records.

Focus groups:
- A semi-structured, pretested questionnaire will be used to explore the provider's perspective about possible barriers and facilitators to screening and second the user perspective discussing factors facilitating or limiting acceptability of self- sampling HPV.

Qualitative interviews of community health workers (CHWs) with the use of a semi-structured pretested questionnaire will be conducted to explore motivation and performance factors of CHWs and discuss recruitment strategies for cervical cancer screening.

6.1.3 Recruitment and screening

The study site will be the health district of Dschang. The Dschang health district is a semi-rural area located in the West Region of Cameroon with an estimated population of 101,385 inhabitants. We evaluated that in the area under study 40,000 women are eligible for CC screening. Recruitment will be done through announcements made in women's associations, churches and integrated health centers (chief nurses of each center will be responsible for this recruitment); announcements will be broadcasted on local radios; and banners will be displayed in hospitals and other public places like markets to inform women of the screening campaign, specifying eligibility criteria, the period and the site of screening. Recruitment of women will also be done by community health workers (CHWs) in their respective "health area". CHWs will be recruited and trained by our study staff. A focus will be made on the free nature of this campaign.

During the screening campaign, the study will be explained to women (information sheet) and their participation will be requested. They will be asked to sign the consent form before the HPV-test. No compensation will be given to the study participants.

6.1.4 Medical chart:

Study data will be collected on personalized electronic and paper medical chart. This will include socio-demographic and medical data (HPV results, cytology results (Pap smear), histologic results (biopsy and ECC), images and videos of the cervix (native, after application of acetic acid and lugol's iodine) and results at follow-up visits.

6.1.5 Sample collection:

Vaginal specimens for HPV test will be collected by participants themselves using flocked swabs after explanations by co-investigators. Two transport mediums will be used for those self-collected vaginal samples: NaCl 0.9%.

Cervical images will be obtained using a Smartphone (Samsung). The first image is that of the native cervix. The second part involves a 2-minute video of 1 frame/second (120 images) captured during application of a 5% acetic acid solution.-The third image is taken following application of Lugol's iodine.

Cervical biopsy will be guided by VIA/VILI if positive. It will be done at 6 o'clock in the transformation zone for participants with negative VIA/VILI. Biopsies will be performed by trained staff using Tischler forceps (Farla medical, United Kingdom).

Pap smear samples will be obtained with a spatula and transported in BD Surepath® vials (Becton Dickinson Company, Franklin Lakes New Jersey, USA) or ThinPrep (Hologic, Bedford, MA, USA). ECC will be done with an endobrush.

6.1.6 Follow-up:

During follow up visit, tests will be done exactly as during the first visit.

6.1.7 AVC development:

A first version of the algorithm will be developed based on videos from VIA and histopathology results only, with a pixel-wise approach, i.e. each pixel will be independently classified. The classification is based on an artificial neural network (ANN) whose inputs and architecture will be optimized. Alternative approaches such as considering spatial or other clinical information will be investigated as well. As the project progresses, the number of data available will increase and the new data will be continuously used to retrain the system to achieve superior performances and generalization capability. Finally, the algorithm will be integrated into a smartphone app, allowing the user to obtain the result and the locations of the lesions immediately after recording the video.

6.1.8 Laboratory analysis:

Dry vaginal swabs will be placed and rinsed into tubes containing either 3 ml of NaCl 0.9% or 3ml PreservCyt® medium and the tubes will be vortexed for 15 seconds. Then 1 ml of each sample will be transferred to the cartridge and will be run on the GeneXpert machine. Once a valid result is obtained, the specimens will be discarded.

GeneXpert HPV analysis: The Xpert HPV analysis consists of real time PCR, using as internal assay control for specimen adequacy, the detection of a human reference gene (HMBS [hydroxymethylbilane synthase]) and an internal Probe Check Control (PCC). The PCC verifies reagent rehydration, PCR tube filling in the cartridge, probe integrity and dye stability.

This test includes reagents for the simultaneous detection of 14 hrHPV types (HPV16, 18, 31, 33, 35, 39, 45, 51, 52, 56, 58, 59, 66 and 68).

The assay utilizes multiple fluorescent channels for the detection of individual types of HPV, groups of HPV, and the human reference gene. Each fluorescent channel has its own cutoff parameters for target detection/validity. If sufficient signal is detected by the human reference gene, the assay results are reported as an overall "positive" if any type of targeted HPV is detected, but, additionally HPV16 and pooled HPV18/45 and, collectively, the other high-risk HPV types detected by the assay are reported specifically as "positive" or "negative."

6.1.9 Methods of minimizing bias

We will use validated pretests questionnaires to register the participants' data and a data manager will check all the database before analysis.

To reduce the number of loss to follow-up, the screen-and-treat strategy will hold in a single day and community agent will help looking for participants necessitating follow up.

7. PROJECT POPULATION

The study setting we would like to implement would have the ability of screening 30 women (30 HPV tests) every day. According to our estimations, 6000 women could be screened during the recruitment period. Women will be recruited in the Dschang health district. (see chapter 4–6.1.3: recruitment and screening).

7.1 Criteria

7.1.1 Inclusion criteria

- Women aged 30-49 years old and able to comply with protocol study
- Women understanding study procedures and accepting voluntarily to participate by signing an informed consent form (ICF).

7.1.2 Exclusion criteria

- Pregnancy
- Previous total hysterectomy
- Conditions that can interfere with visualization of the cervix

7.1.3 Criteria for withdrawal / discontinuation of participants

The participants can decide to withdraw from the study at any moment. We will explain to them that all medical and biological data obtained up until then will be used for the purposes of the study. In case of disease progression, patients will be managed according to the WHO guidelines for screening and treatment of precancerous lesions for cervical cancer prevention (Organization, 2013).

8. PROJECT ASSESSMENTS

8.1 Project flow chart/ table of procedures and assessments

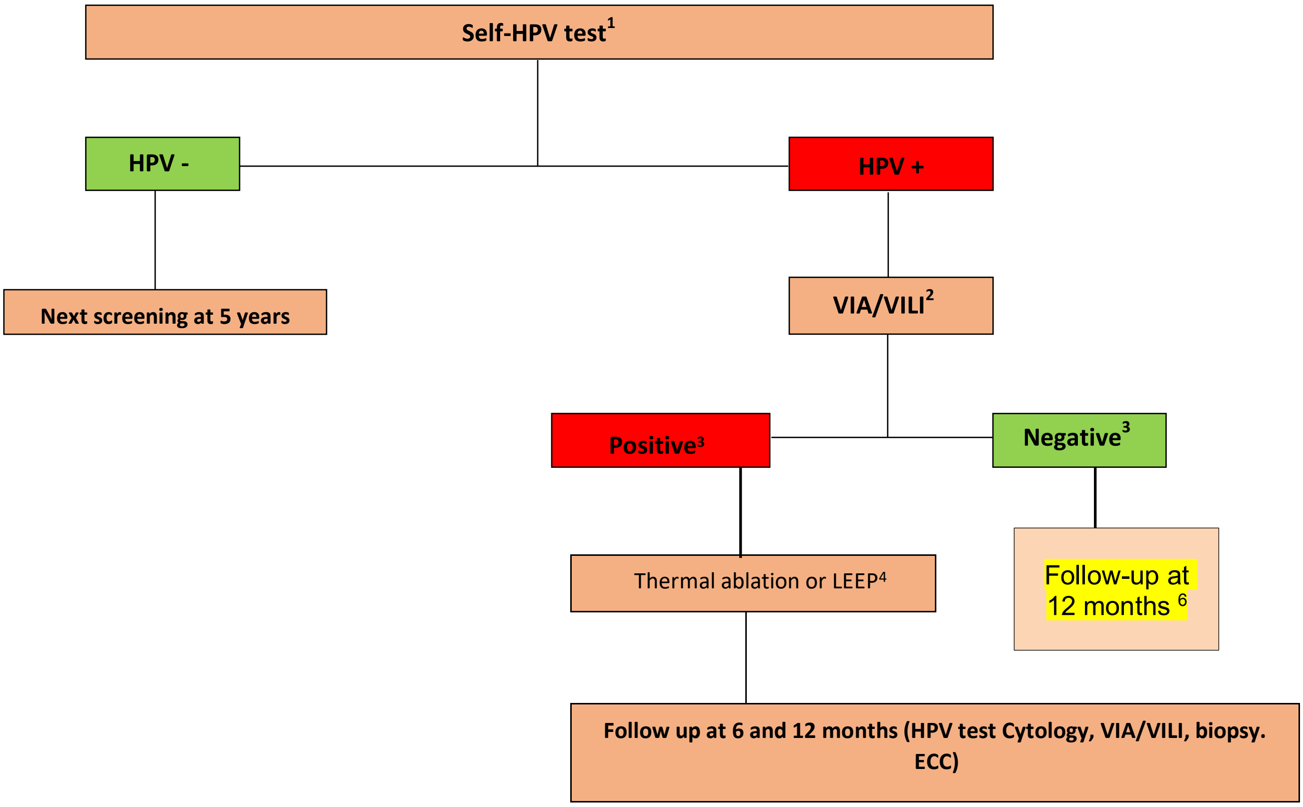

Figure 1. Flow chart of the study

[1]Use of GeneXpert® test; [2]If suspicious of cancer refer to tertiary center; [3] All HPV positive women will have as quality control a cytology and guided biopsy (or random biopsy if VIA/VILI normal); a recall will be done if abnormal cytology/histology in untreated women. [4]LEEP (Loop electrosurgical Excisional Procedure) in case of ineligibility to Thermal ablation. HPV: Human papillomavirus; LEEP: Loop electrosurgical excision procedure; VIA: Visual inspection with acetic acid; VILI: Visual inspection with Lugol's iodine

8.2 Assessments of primary endpoint/ outcome

Sensitivity, specificity, positive predictive value and negative predictive value of VIA/VILI to detect CIN2+ lesions among HPV-positive women be calculated, using histology as gold standard.

8.3 Assessment of secondary endpoint/ outcomes

In the conditions cited above, women with suspected precancerous lesions following VIA/VILI, will be immediately treated. The personnel will be trained to recognize fistulas. Patients with a fistula, will be transferred to the University Teaching Hospital of Yaoundé and will be treated free of charge. Voluntary women will be addressed to the HIV department for screening and free treatment. The feasibility of a prevention program for cervical cancer, sexually transmitted diseases and obstetrical fistula, will be assessed by the local teams in Cameroon and the University hospital of Geneva.

The University Hospital of Geneva will invest in the development of a consultation room in the district hospital of Dschang, with the purchase of a gynecologic table, a colposcope and initial renewable material. The GeneXpert® machine is already available in Dschang. Geneva will also insure the training of the medical team including nurses, one medical technician and CHWs, and the salary and/or financial compensations of this team during the 72-month study period. This team will then be supervised by a part-time gynecologist.

At the end, an activity report will allow us to evaluate the efficiency of the advertisement campaign, the number of screened women, and ability of the Ministry of Health to take over the fees of the material and human resources to carry on with the promotion of women health.

8.4 Assessment of other study variables

Socio-demographics and past obstetric and gynecological history will be assessed during collection at the first screening visit.

At the end, the extrapolation of our data, will give us an idea of the prevalence of HPV infection and cervical pre-cancer and cancer among Cameroonian women.

8.5 Assessment of safety and reporting

8.5.1 Definition of Serious Events (SEs)

A serious event is any unfavorable event for which a causal relationship to sampling of biological material or the collection of health related personal data cannot be ruled out, and which:

- requires hospitalization or prolongation of an inpatients' hospitalization,
- results in persistent or significant disability or incapacity, or
- is life-threatening or results in death, If a serious event occurs the research project will be set on hold.

8.5.2 Assessment and Documentation of SEs

Application and interpretation of the VIA results will be conducted as recommended by the WHO. « Relaxed » VIA criteria will be used, meaning that any whitening of the cervix after acetic acid application will be considered as positive (to reduce false negative rate). Application of acetic acid on the cervix may cause a slight burning sensation, which disappears after a few minutes. The application of iodine does not cause any discomfort if not a change in color of the cervical surface, but the normal color returns a few minutes after the procedure. As for the cell and tissue specimens, minor pain and/or bleeding may be observed after sampling. But most often, these inconveniences are harmless and disappear within hours after the procedure. All the SEs will be documented in the participants' file and on the SE report form.

8.5.3 Reporting of SAEs, Safety and Protective Measures

Reporting of SAEs

All SAEs will be reported immediately and within a maximum of 24 hours to the Sponsor- Investigator of the study. The Sponsor-Investigator will re-evaluate the SAE and return the form to the site. SAEs resulting in death will be reported to the local Ethics Committee (via local Investigator) within 7 days.

Reporting of Suspected Unexpected Serious Adverse Reactions (SUSARs)

A SUSAR will to be reported to the local Ethics Committee (local event via local Investigator) within 7 days, if the event is fatal, or within 15 days (all other events).

Reporting of Safety Signals

All suspected new risks and relevant new aspects of known adverse reactions that require safety-related measures, i.e. so called safety signals, must be reported to the Sponsor- Investigator within 24 hours. The Sponsor-Investigator must report the safety signals within 7 days to the local Ethics Committee (local event via local Investigator).

Reporting and Handling of Pregnancies

Our study will not include pregnant participants. Any pregnancy occurring during the study will be reported to the Sponsor-Investigator within 24 hours. Those women found with CIN2+ will be followed throughout the pregnancy and six weeks after birth evaluation and the treatment of the CIN2+ will be offered to them. Those normal cytology and histology results will been seen at the follow visit six weeks after delivery for HPV-test, Pap smear and ECC. All participants exiting the study due to a SAE will be monitored until the complete resolution of symptoms and signs. Efforts will be made in order to minimize any loss to follow-up, through supervision by the most experienced local physician.

9. STATISTICAL METHODOLOGY

9.1 Determination of Sample Size

We plan to include 6000 women in the study. Assuming a prevalence of HPV infection of 20%, 1200 participants will be HPV+. Among the awaited 1200 HPV+ women, 120 will have CIN2+ lesions (assuming a prevalence of CIN2+ of 10% among HPV+ women). Assuming a 20% drop-out rate, 96 cases of CIN2+ will be analyzed to determine the sensitivity of VIA (expected around 60%) with a precision of +/- 9.8%.

Among the awaited 1200 HPV+ women, 1080 will not have CIN2+ lesions. With a drop-out rate of 20%, 864 cases of non-CIN2+ will be analyzed to determine the specificity of VIA (expected around 50%) with a precision of +/- 3.3%.

9.2 Data processing

Study data will be recorded with paper Case Report Forms (p-CRF). The p-CRF will be coded with the patient’s number of inclusion in the study. Only the study’s investigators will be authorized to enter data in the p-CRF. Data from the p-CRFs will then be transferred into an electronic database for analysis.

Some data will be collected directly in the p-CRF because in the study areas there are no complete medical files. This will include socio-demographic and medical parameters, visit dates, randomization number, SAEs and test results. Nevertheless, some tests results (HPV- test results, cytology and histology reports) will be available in laboratories as source data.

Data entry from the p-CRF into the electronic database using Secutrial® software will be done during the study. At the end of the study, all data will be double checked in order to guarantee accuracy. Any discordance will be clarified by calling the participant. All data in the p-CRF and in the electronic database will only refer to patients by their anonymity number.

The coded data used for the development of the AVC test will be stored on a Secure File Server (NAS) accessible only to the authorized personnel with a personalized authentication system.

9.3 Planned analysis

9.3.1 Datasets to be analyzed

Data from all complete electronic CRFs will be analysed. We will carry out our analyses using the software package STATA® 14 (Stata, College Station, TX, USA). All p-values of <0.05 will be considered statistically significant. Sensitivity, specificity, positive and negative predictive values of VIA/VILI will be calculated using histology as gold standard. The aforementioned trial statisticians will perform these analyses.

Qualitative interviews (e.g. focus groups discussions or individual interviews) will be recorded, and

transcribed. The transcripts will be analyzed using the software ATLAS.ti.CAQDAS or manually applying the qualitative content analysisaccording to Mayring. [11]

9.3.2 Handling of missing data

Missing data will be reported as such. Drop-outs will be replaced.

9.3.3 Deviations from the original statistical plan

Any deviations from the planned analyses will be justified and reported in the final manuscript.

10. DATA AND QUALITY MANAGEMENT

10.1 Data handling and record keeping / archiving

Each participant's signed informed consent form and personal information (including the socio-demographic questionnaires and the post-treatment assessment) will be classified by participant anonymity number and stored in a closet to which only the designed medical personnel will have access through the use of key. Prior to the beginning of the study (enrollment of participants) the principal investigator will organize a training session for all the study personnel. Copies of all the study documents will be kept study master file secured at the study in a safe while originals will be kept by the sponsor-principal investigator. Access to that safe will be limited to the site coordinating investigator and to designated co-investigator. These documents include (source/data documents): ethical and administrative clearances, advertising messages, anonymity list, participants information sheets with signed informed consent, pCRFs, results of HPV test, cytological and histological analyses, electronic drives and smartphones containing pictures of the cervix, records of adverse reactions, curriculum vitae of study personnel and reports of monitoring visits and audits).

Relevant clinical information along with the videos taken during VIA will be transferred to EPFL in order to develop an AVC tool. Only coded data is transferred.

10.2 Data transfer

Exports of all or any kind of partial data will be systematically password-protected before being transferred and only coded data will be used.

Data from the p-CRF is entered into the electronic database using Secutrial® software in Cameroon and does not necessitates any transfer to be accessible from HUG.

The native/VIA/VILI photos and VIA videos are taken with a password-protected smartphone that is only dedicated for that purpose. A mobile application is being specifically developed to collect the images and to securely transfer them to a secure file server (NAS) at EPFL via an API hosted on a private EPFL server, using HTTPS.

10.3 Coding and back-up

Study data will be recorded with paper Case Report Forms (p-CRF). The p-CRF will be coded with the patient's number of inclusion in the study. The code may only be broken if it is necessary to avert an immediate risk to the health of the person concerned or to guarantee the rights of the person or a legal basis exists for breaking the code. Bi-weekly check-up, electronic validation and back-up of datasets (obtained from e-CRFs) will be done by thedata manager and site-coordinator.

10.4 Confidentiality, Data Protection

Direct access to source documents will be allowed for purposes of audits and inspections. The Cameroonian national ethics committee may have access to protocol, dataset, statistical code and other information during the study. The results of the study will be published in a scientific peer-review journal while maintaining the patients' information strictly confidential.

10.5 Archiving and Destruction

All study data (consent forms, pCRF, eCRF, datasets, drives with cervical images, anonymity codes,

ethical and administrative clearances, monitoring and audits/inspections reports), cytology and histology samples will be archived for a minimum of 10 years after study termination or premature termination of the study in the laboratory of clinical research of the Gynecology division of the Geneva University Hospitals. All HPV-test samples will be destroyed at the end of the study.

10.6 Monitoring and audits/inspections

The Sponsor-Principal Investigator will organize initial, follow-up and closing monitoring visits (to be carried out by himself, by Professor Pierre Vassilakos, by Pr Pierre Marie Tebeu and Pr Zacharie Sando). During these visits all the study documents (source and data) and will be made accessible to the monitor and all the co-investigator will answer all the questions. In case of audit/inspection visit by competent authorities all the study personnel will answer all the questions and all the data/source documents will be made available.

Access to the database may be done at any time by the primary investigator as well as the co-investigators for study monitoring and quality control.

11. PUBLICATION AND DISSEMINATION POLICY

11.1 Publication of results

The results of the study, (positive of negative) will be submitted for publication in a peer- reviewed journal and will be presented at scientific congresses in Cameroon, Switzerland and elsewhere. Study report will also be given to Cameroonian health authorities. Participants will receive the reports via their health staff at their respective medical facilities in the Dschang and health district. The project data can be accessed by third parties by contacting the methodologist of the study.

11.2 FUNDING AND SUPPORT

Financial support has been received from the University Hospitals of Geneva (*Commission des Affaires Humanitaires*), *Solidarité Internationale* of Geneva, the GRSSGO (groupement romand de la société Suisse de gynécologie et obstétrique), Tech4Dev program from EPFL, and Swiss Cancer League. The funders in the research project will help the financing of the initial set-up of this prevention program (material resources, training and salary of human resources).

11.3 INSURANCE

The study will be carried out in two district hospitals in Cameroon which has adequate infrastructures and qualified human resources to ensure appropriate management of any side effects, or complication that may arise during the study's procedure. Arrangements will be made in Cameroon for management of these complications totally free of charge for participants.

12. REFERENCES

1. *American Cancer Society. Global Cancer Facts & Figures 2nd Edition. .* Atlanta: American Cancer Society, 2011.
2. Arbyn, M., et al., *Evidence regarding human papillomavirus testing in secondary prevention of cervical cancer.* Vaccine, 2012. **30**: p. F88-F99.
3. Ronco, G., et al., *Efficacy of HPV-based screening for prevention of invasive cervical cancer: follow-up of four European randomised controlled trials.* The lancet, 2014. **383**(9916): p. 524-532.
4. Arbyn, M., et al., *Accuracy of human papillomavirus testing on self-collected versus clinician-collected samples: a meta-analysis.* The lancet oncology, 2014. **15**(2): p. 172-183.
5. Sherris, J., et al., *Evidence-based, alternative cervical cancer screening approaches in low-resource settings.* International perspectives on sexual and reproductive health, 2009: p. 147-152.
6. Sankaranarayanan, R., et al., *HPV screening for cervical cancer in rural India.* New England Journal of Medicine, 2009. **360**(14): p. 1385-1394.
7. Einstein, M.H., et al., *Clinical evaluation of the cartridge-based GeneXpert human papillomavirus assay in women referred for colposcopy.* Journal of clinical microbiology, 2014. **52**(6): p. 2089-2095.
8. Chibwesha, C.J., et al., *Clinical Performance Validation of 4 Point-of-Care Cervical Cancer Screening Tests in HIV-Infected Women in Zambia.* Journal of lower genital tract disease, 2016.
9. Toliman, P., et al., *Field evaluation of the Xpert® HPV Point of Care Test for the detection of human papillomavirus infection using self-collected vaginal and clinician-collected cervical specimens.* Journal of clinical microbiology, 2016: p. JCM. 00529-16.
10. Eperon, I., et al., *Randomized comparison of vaginal self-sampling by standard vs. dry swabs for Human papillomavirus testing.* BMC cancer, 2013. 13(1): p. 353.
11. J. Bigoni et al., “Cervical cancer screening in sub-Saharan Africa: A randomized trial of VIA versus cytology for triage of HPV-positive women,” Int. J. Cancer, vol. 137, no. 1, pp. 127–134, Jul. 2015.
12. P. E. Gravitt et al., “Effectiveness of VIA, pap, and HPV DNA testing in a cervical cancer screening program in a Peri-Urban community in Andhra Pradesh, India,” PLoS One, vol. 5, no. 10, 2010.
13. K. O. Ajenifuja et al., “A population-based study of visual inspection with acetic acid (VIA) for cervical screening in rural nigeria,” Int. J. Gynecol. Cancer, vol. 23, no. 3, pp. 507–512, Mar. 2013.
14. I. J. Etherington, D. M. Luesley, M. I. Shafi, J. Dunn, L. Hiller, and J. A. Jordan, “Observer variability among colposcopists from the West Midlands region,” BJOG An Int. J. Obstet. Gynaecol., vol. 104, no. 12, pp. 1380–1384, 1997.
15. P. M. Tebeu et al., “Effectiveness of a two-stage strategy with HPV testing followed by visual inspection with acetic acid for cervical cancer screening in a low-income setting,” Int. J. Cancer, vol. 136, no. 6, pp. E743–E750, Mar. 2015.
16. M. Kunckler et al., “Cervical cancer screening in a low-resource setting: a pilot study on an HPV-based screen-and-treat approach,” Cancer Med., vol. 6, no. 7, pp. 1752–1761, Jul. 2017.
17. M. A. Devi, S. Ravi, J. Vaishnavi, and S. Punitha, “Classification of Cervical Cancer Using Artificial Neural Networks,” in Procedia Computer Science, 2016, vol. 89, pp. 465–472.
18. B. Sokouti, S. Haghipour, and A. D. Tabrizi, “A framework for diagnosing cervical cancer disease based on feedforward MLP neural network and ThinPrep histopathological cell image features,” Neural Comput. Appl., vol. 24, no. 1, pp. 221–232, Jan. 2014.
19. M. Wu, C. Yan, H. Liu, Q. Liu, and Y. Yin, “Automatic classification of cervical cancer from cytological images by using convolutional neural network,” Biosci. Rep., vol. 38, no. 6, p. 20181769, Nov. 2018.
20. B. Taha, J. Dias, and N. Werghi, “Classification of cervical-cancer using pap-smear images: A convolutional neural network approach,” in Communications in Computer and Information Science, 2017, vol. 723, pp. 261–272.

21. E. J. Mariarputham and A. Stephen, “Nominated texture based cervical cancer classification,” Comput. Math. Methods Med., vol. 2015, Jan. 2015.
22. H. A. Almubarak et al., “Convolutional Neural Network Based Localized Classification of Uterine Cervical Cancer Digital Histology Images.,” in Procedia Computer Science, 2017, vol. 114, pp. 281–287.
23. J. Hyeon, H. J. Choi, B. D. Lee, and K. N. Lee, “Diagnosing cervical cell images using pre-trained convolutional neural network as feature extractor,” in 2017 IEEE International Conference on Big Data and Smart Computing, BigComp 2017, 2017, pp. 390–393.
24. Y. Xiang, W. Sun, C. Pan, M. Yan, Z. Yin, and Y. Liang, “A novel automation-assisted cervical cancer reading method based on convolutional neural network,” Biocybern. Biomed. Eng., vol. 40, no. 2, pp. 611–623, Apr. 2020.
25. Mayring, Philipp (2000), *Qualitative Content Analysis.* Forum Qualitative Sozialforschung / Forum: Qualitative Social Research, [S.l.], v. 1, n. 2, june 2000. ISSN 1438-5627. doi:http://dx.doi.org/10.17169/fqs-1.2.1089.
